# Evaluating antioxidant treatment approaches for eosinophilic chronic sinusitis

**DOI:** 10.3389/fimmu.2026.1780245

**Published:** 2026-06-18

**Authors:** Takeshi Kusunoki, Davis Joseph, Katsuhisa Ikeda, Fukka You, Haruhiko Inufusa

**Affiliations:** 1 Department of Otorhinolaryngology, Juntendo University of Medicine, Shizuoka Hospital, Tokyo, Japan; 2 Division of Antioxidant Research, Life Science Research Center, Gifu University, Gifu, Japan; 3 Flogen Technologies Inc., Mount Royal, QC, Canada; 4 FLOGEN Technologies Inc., Wilmington, DE, United States; 5 Department of Otorhinolaryngology, Juntendo Tokyo Koto Geriatric Medical Center, Tokyo, Japan; 6 Anti-oxidant Research Laboratory, Louis Pasteur Center for Medical Research, Kyoto, Japan

**Keywords:** antibody, antioxidant, AP-1, B cell, C/EBP δ, carbon monoxide, catalase, claudin-4

## Abstract

Chronic rhinosinusitis with eosinophilia (ECRS) is a refractory sinusitis characterized by eosinophilia in the nasal mucosa and peripheral blood. In ECRS, multiple nasal polyps are present in the ethmoid sinuses, and the disease is often accompanied by olfactory dysfunction and asthma. It is a disease that significantly impairs the quality of life of patients by causing persistent nasal congestion and thick nasal discharge, leading to decreased concentration and insomnia. From previous studies, we found that (1) defects in Cu,Zn-SOD in ECRS epithelium contribute to increased IL-17A, macrophage infiltration in the subepithelial tissue, and excessive production of mucin gene (MUC5AC) in the epithelium, thereby potentially exacerbating inflammation and excessive mucus secretion, and (2) reduced HO-1 expression in the epithelium and macrophage infiltration are associated with epithelial damage in CRS accompanied by eosinophil infiltration. These findings suggest that antioxidants may play a crucial role in elucidating the pathophysiology of refractory diseases such as ECRS and may provide new therapeutic strategies. In this paper, three new comprehensive molecular signaling pathway networks of (1) oxidative stress, (2) inflammation inhibition mechanisms and (3) inflammation in ECRS were developed for the first time, based on a critical analysis of scientific literature. In addition, three immunology flowsheets related to oxidative stress, inflammation and carbon monoxide-based inflammation inhibition mechanisms of ECRS were developed for the first time and examined from both clinical and biochemical perspectives. Based on these pathways, this study identified the treatment targets which can be used in future ECRS treatments.

## 1 Introduction

Chronic rhinosinusitis (CRS) is defined as persistent inflammation of the nasal and paranasal mucosa for a minimum of three months (1). Since the paranasal sinuses are adjacent to the orbital and cranial cavities, inflammation from acute sinusitis or acute exacerbations of chronic sinusitis can spread to these cavities, leading to severe complications. Given that the underlying causes of CRS can vary, individualized treatment strategies are required rather than a one-size-fits-all approach (2, 3). Recently, Japan has seen an increasing trend of eosinophilic chronic rhinosinusitis (ECRS), which is resistant to drug therapy and surgery (4). ECRS is a condition characterized by an increase in eosinophils in peripheral blood and numerous eosinophil infiltrates in the nasal mucosa and nasal polyps. Patients primarily affected by nasal obstruction and olfactory dysfunction often have multiple bilateral nasal polyps. This leads to a significant decline in quality of life and substantial social burden. To promote the diagnosis and treatment of ECRS, the Ministry of Health, Labor and Welfare conducted a research project on intractable diseases titled “Japanese Epidemiological Survey of Refractory Eosinophilic Chronic Rhinosinusitis Study”. This project resulted in the establishment of diagnostic criteria known as JESREC (**Table 1**).

**Table 1:** Criteria for diagnosis of eosinophilic chronic rhinosinusitis (JESREC score).

Factor	Criterion	Score
Bilateral Involvement	Both sides affected	3
Nasal Polyps	Presence of polyps	2
CT Findings	Ethmoid dominance (Ethmoid/Maxillary ratio $\ge 1$)	2
Peripheral Blood Eosinophils (%)	Between 2% and 5%	4
	Between 5% and 10%	8
	Higher than 10%	10

When the total score is 11 points or higher, the possibility of eosinophilic chronic rhinosinusitis is high. A definitive diagnosis of eosinophilic chronic rhinosinusitis should be made by microscopic examination of nasal polyp/paranasal sinus tissues, if three fields inspected under a microscope at a total magnification of 400 (ocular lens, field number 22) show 70 or more eosinophils per field.

In Japan, ECRS is classified as an intractable disease due to its challenging management in clinical settings (5), and it is also recognized worldwide as a difficult-to-treat disease. ECRS is classified as a type 2 inflammatory disease due to its similarities in pathogenesis with type 2 diseases such as bronchial asthma and atopic dermatitis, and is often associated with them. A type 2 inflammatory disease is a disease involving an immune response from Th2 helper cells and the production of immunoglobulin E (IgE) antibodies by B cells. ECRS is known to have a high prevalence rate in conjunction with asthma (2, 4, 5), and cases with asthma are classified as moderate or severe, with a high recurrence rate, according to the JESREC scoring system. If ECRS is present, the possibility of bronchial asthma should also be considered. In such cases, since the nasal cavity and bronchi are connected, it is important to consider them as a single airway, because treating airway symptoms in the upper or lower respiratory tract can help improve symptoms in other tract. Chen et al. (6), Poston et al. (7) and Seminario et al. (8) have reported that the infiltration of macrophages and eosinophils is closely linked to the severity of asthma. Free radicals, which are produced by inflammatory cells such as macrophages, have been shown by Vendrov et al. (9) to impact both the lungs and exacerbation of intractable sinusitis by contributing to airway inflammation. Additionally, changes in the levels of antioxidants, which neutralize free radicals, have been associated, as per Iijima et al. (10), with the progression of nasal and paranasal diseases. Furthermore, as described by Makihara et al. (11), interleukin-17A (IL-17A), a cytokine associated with macrophages, has been implicated in ECRS pathogenesis. Nishi et al. (12) reported that IL-17A promotes MUC5AC gene expression, leading to the production of viscous nasal discharge, a hallmark of ECRS. Staitoh et al. (13), Saitoh et al. (14) and Ono et al. (15), have previously reported that eosinophils, macrophages, IL-17A, and MUC5AC genes serve as key exacerbating factors in ECRS. In Ono et al. (16) and Kusunoki et al. (17), it was reported that antioxidants could suppress these exacerbating factors. in the context of ECRS, two well-known antoxidants, superoxide dismutase (SOD) and heme oxygenase-1(HO-1), have been extensively studied. SOD research, in particular, has been widely applied in clinical studies across various institutions. In Ono et al. (15), the causal link between antioxidants and proinflammatory factors involved in ECRS was established by performing endoscopic sinus surgery to collect nasal tissue samples of eosinophilic sinusitis (ECRS) and non-eosinophilic sinusitis (non-ECRS) that were sectioned (3.5 μm) and subjected to immunohistochemical staining for macrophages (CD68), MUC5AC, IL-17A, Cu, Zn-SOD, and HO-1, as well as by measuring the mRNA expression of SOD. In this paper, we analyze the relationship between antioxidant substances (SOD, HO-1) and the main aggravating factors of ECRS (eosinophils, macrophages, IL-17A, MUC5AC) by examining previous related literature. Based on this, we develop the comprehensive molecular signaling networks involving ECRS, inflammation, the hypoxia inducible factor-1 (HIF-1), the protein tyrosine phosphatase non-receptor type 2 (PTPN2), the nucleotide-binding domain, leucine-rich–containing family, pyrin domain–containing-3 (NLRP3) inflammasome, SOD, and B cell immunoglobulin production. Using these developed networks as a template, we identify the treatment targets that should be emphasized in future ECRS treatment.

## 2 The role of IL-17A in ECRS

In ECRS, eosinophil infiltration is associated with severe epithelial damage. The secretion of certain proteinases and cytokines by eosinophils can lead to airway epithelial remodeling, such as epithelial detachment and basement membrane thickening (18, 19). Ono et al. (15) demonstrated that the pathogenesis of ECRS is associated with the mobilization of macrophages, and that there is a significant increase in the number of macrophages (CD68-positive cells) and IL-17A-positive cells in patient tissue with ECRS compared to non-ECRS patients and control groups. Additionally, the percentage of epithelial cells with a positive MUC5AC reaction was significantly higher in ECRS patients than in non-ECRS patients or in control groups. Significant positive correlations were found between CD68 and IL-17A-positive cells in ECRS, as well as between the percentage of epithelial cells with MUC5AC-positive reactions and IL-17A-positive cells.

Even in asthma, which frequently occurs concomitantly with ECRS, eosinophils are recognized as key mediators in the pathology and abnormal physiology of asthma (8). Matrix metalloproteinases (MMPs) play a role in airway remodeling in asthma (18). In severe asthma, macrophages (7, 20) and macrophage-derived MMPs are elevated (19, 21). Moreover, some studies suggest that macrophages are involved in the production of IL-17A (6). Mucus hypersecretion and persistent airway inflammation are consequences of increased MUC5AC expression. IL-17A can induce MUC5AC expression in the airways (1). Endobronchial tissues in severe asthma exhibit increased macrophage infiltration (20), and these macrophages can produce oxidants (9). Ijima et al. (10) demonstrated that oxidants exacerbate nasal allergy-like symptoms by inducing nasal hyperresponsiveness and eosinophil infiltration. Poston et al. (7) reported that, in patients with asthma, the total number of macrophages infiltrating the airway mucosa was elevated and suggested that lung macrophages play a central role in the chronic immune-mediated inflammatory response in the airway mucosa of asthmatic patients. In Saitoh et al. and Ono et al. (13, 15), we examined whether severe epithelial damage is associated with infiltrating macrophages and eosinophils in ECRS. The results showed that the number of macrophages with CD68-positive reactions in the subepithelia was significantly higher in ECRS compared to non-ECRS. As per Vendrov et al. (9) and Wenzel (20), macrophages and eosinophils secrete various cytotoxic factors, such as oxidants and proteinases, which may contribute to severe epithelial damage in ECRS. Furthermore, a significant increase in the number of IL-17A-positive cells was observed in ECRS compared to non-ECRS. Immunohistochemical analysis demonstrated IL-17A expression in the presence of infiltrating inflammatory cells. Makihara et al. (11) reported that IL-17A expression in CRS is detectable in eosinophils, macrophages, and lymphocytes through double staining. In Saitoh et al. (14), a significant correlation was reported between eosinophils and IL-17A-positive cells, as well as between macrophages and IL-17A-positive cells. Therefore, eosinophils and macrophages likely serve as the primary producers of IL-17A in ECRS. A summary of these findings is presented in **Table 2**.

**Table 2:** Exacerbating factors of eosinophilic sinusitis (ECRS) pathology.

Comparison/Measurement	ECRS vs Non-ECRS	Statistical Significance
Number of CD68 (macrophage) positive cells	ECRS > Non-ECRS	P < 0.001
Number of MUC5AC positive cells	ECRS > Non-ECRS	P < 0.05
Number of IL-17A positive cells	ECRS > Non-ECRS	P < 0.0001
IL-17A positive cells vs CD68 (macrophage) positive cells	Positive correlation	P < 0.01
IL-17A positive cells vs MUC5AC positive cells	Positive correlation	P < 0.05

For the treatment of eosinophilic sinusitis, it is necessary to suppress not only eosinophils but also macrophages and related cytokines (IL-17).

It was found that the degree of epithelial damage and basement membrane thickness was dependent on the number of infiltrating eosinophils and IL-17A-positive cells. Chen et al. (6) reported that IL-17A could induce mucin gene expression, such as MUC5AC, as well as eosinophil infiltration in the airways. In Saitoh et al. (14), a significant increase in the mean number of IL-17A-positive cells was observed in ECRS compared to non-ECRS. There was a significant correlation between MUC5AC and IL-17A-positive cells. IL-17A in the subepithelial layer may stimulate MUC5AC expression in the epithelial cells of ECRS, similar to its effect in the airways.

In recent years, the concept of sinusitis in Japan has recently shifted from the traditional phenotype classification, which divides cases based on the presence or absence of nasal polyps, to a more refined classification based on specific mechanisms and molecular biomarkers. This change reflects the growing understanding of molecular-targeting research. As per Sawatsubashi et al. (22), three primary inflammatory endotypes have been established, significantly altering the pathological concepts and treatment approaches. As described by Fokkens et al. (23), Haruna et al. (24) and Tokunaga et al. (25), ECRS belongs to type 2 inflammatory chronic sinusitis and often has characteristic clinical manifestations that are different from bacterial chronic sinusitis, which is the conventional type I inflammatory sinusitis. Each of the three types has different inflammatory cells, cytokines, and mechanisms of inflammation, which are the center of the pathological condition, and the concept of the disease is also very different. Additionally, it has been reported by Giunta et al. (26), Cho et al. (27), Akdis et al. (28), Bachert et al. (29) and Bachert et al. (30) that the treatment method differs depending on the inflammatory endotype classification of chronic sinusitis. **Table 3** shows three inflammatory endotypes in chronic sinusitis, based on past literature by Haruna et al. (24), Tokunaga et al. (25), Giunta et al. (26), Cho et al. (27) and Bachert et al. (29). The main cytokines in the type 1 inflammatory response are IFN-γ, which involve Innate lymphoid cell 1 (ILC1), type I helperT cells (Th1 cells), neutrophils, and macrophages. The main cytokines in the type 2 inflammatory response are IL-4, IL-5, and IL-13, and the reaction is centered on helper T cell type 2 (Th2), ILC2, and eosinophils. In the type 3 inflammatory response, the main cytokines are IL-17A and IL-22, and main inflammatory cells are Innate ILC3 and Th 17 cells. As per Sawatsubashi et al. (22), these inflammatory response pathways are chronically activated in the sinus tissue by a combination of one or more responses. A typical disease of type 2 inflammatory sinusitis is eosinophilic sinusitis. However, according to our study, macrophages, the main inflammatory cells of type 1 inflammation, and IL-17A, the main cytokine of type 3 inflammation, were significantly more abundant in the nasal mushroom tissue of ECRS than non-ECRS. These results suggest that eosinophilic sinusitis may be affected not only by type 2 inflammation but also by type 1 and type 3 endotypes.

**Table 3:** Inflammatory types of chronic rhinosinusitis.

Feature	Non-ECRS	ECRS
Dominant Cell Type	Neutrophils	Eosinophils
Macrophage Infiltration	Low (Minimal CD68+ cells)	High (Significant CD68+ cells)
Cytokine Profile	Primarily Type 1/Type 17	Dominant Type 2/Type 17 (↑ IL-17A, IL-5)
Antioxidant Expression	Normal SOD & HO-1 levels	Deficient (↓ Cu,Zn-SOD & ↓ HO-1)
Epithelial Integrity	Intact Tight Junctions	Disrupted (↓ Claudin-4, Occludin, ZO-1)
Mucus Production	Normal viscosity	Hypersecretion (↑ MUC5AC, highly viscous)
Tissue Remodeling	Minimal	Severe (Basement membrane thickening, polyposis)
Clinical Presentation	Moderate obstruction	Anosmia, polyps, and frequent asthma comorbidity
Steroid Sensitivity	Generally Responsive	Frequent Resistance (Linked to NLRP3 activation)

From the above, an important cytokine (signaling protein for immune cells) affecting ECRS can be confirmed as IL-17A (also known as IL-17), produced primarily by T_H_17 cells, macrophages and eosinophiles. As per Jovanovic et al. (31), IL-17A induces the accumulation and binding to DNA of the following transcription factors: (a) the nuclear factor kappa B (NF-κB), (b) the activator protein (AP-1), and (c) the cyclic AMP response element binding protein (CREB). When these transcription factors bind to enhancer/promoter sequences found in the genes of DNA, they increase the expression of inflammation inducing cytokines produced by macrophages such as IL-1β, TNF-α, and IL-6.

As per Rahman et al. (32), IL-17A also contributes to inflammation by increasing the production of eotaxin-1, also known as CCL11. As per Ivanovska et al. (33), eotaxin-1 is a cytokine which attracts eosinophile immune cells to inflammatory sites so they can contribute to the inflammation process. Furthermore, as per Rahman et al. (32), IL-17A causes eotaxin-1 production through mitogen-activated protein kinases (MAPKs), which are enzymes that phosphorylate proteins. As proof, Rahman et al. (32) found that IL-17A-induced eotaxin-1 production was significantly reduced when inhibition of the following MAPKs occurred: (a) p38, (b) p42, (c) p44 extracellular signal-regulated kinases (ERK), (d) c-Jun N-terminal kinase (JNK), and (e) the Janus kinase (JAK).

Huang et al. (34) studied inflammation in experimental autoimmune encephalomyelitis (EAE), an immune disease with signaling pathways identical to ECRS. They observed that the inflammatory response caused by IL-17A was impaired in mice when they deleted the gene (MAPK14) that encodes the production of p38α, a MAP kinase (MAPK). This is because the MAPK-activated protein Kinase 2 (MK2), which activates the production of inflammatory proteins, cannot be activated through phosphorylation when mice lack MAPK. Moreover, as per Huang et al. (34), MAPK gene deletion and direct MK2 gene inhibition both resulted in a significant reduction in the production of pro-inflammatory proteins. As a result of MK2 activity impairment, immune cells like T_H_17 cannot effectively infiltrate inflammatory sites and initiate the inflammatory response.

As per Huang et al. (34), CXCL1’s production is reduced upon MAPK gene deletion. CXCL1 is a chemokine responsible for the attraction of neutrophils, a subtype of immune cells, to inflammatory sites (35). However, Huang et al. (34) reported that CXCL1 was overexpressed when mitogen-activated protein kinase phosphatase 1 (MKP-1), an inhibitor of MAPK activity, was deficient in cells. Tierney et al. (36) confirmed that the production of eotaxin-1 (CCL11) decreased after MK2 inhibition, same as CXCL1. Altogether, this study concludes that these investigations demonstrate the importance of IL-17A in the activation and production of multiple transcription factors and kinases involved in the molecular signaling pathways of the type 3 inflammatory response.

Based on this critical analysis of the aforementioned scientific literature, it is concluded that since MAPK activity inhibition impairs IL-17A signaling and reduces the inflammatory response, MAPK inhibitors can be a potential therapeutic option for ECRS to mitigate the chronic inflammatory response. There are also other therapeutic targets related to IL-17A and ECRS that are listed below:

- MAP Kinases
- Eotaxin-1
- CXCL1
- MAPK-activated protein kinases

Based on this critical analysis of the above-mentioned scientific literature, several biochemical flowsheets were developed in this paper, and the signaling pathways of this section are shown in some specific parts of each figure, along with many other signaling pathways.

## 3 The role of SOD and catalase in ECRS

Tai et al. (37) pointed out that excessive production of reactive oxygen species (ROS) in CRS causes oxidative stress (OS). Oxidative stress is crucial in regulating the immune response and can serve as a pathogenic factor in various diseases. CRS is a complex, heterogeneous disease with multiple phenotypes and endotypes. Recent studies have increasingly suggested that oxidative stress, induced by both environmental and intrinsic stimuli, plays an important role in the pathogenesis and persistence of CRS.

**Figure 1:**
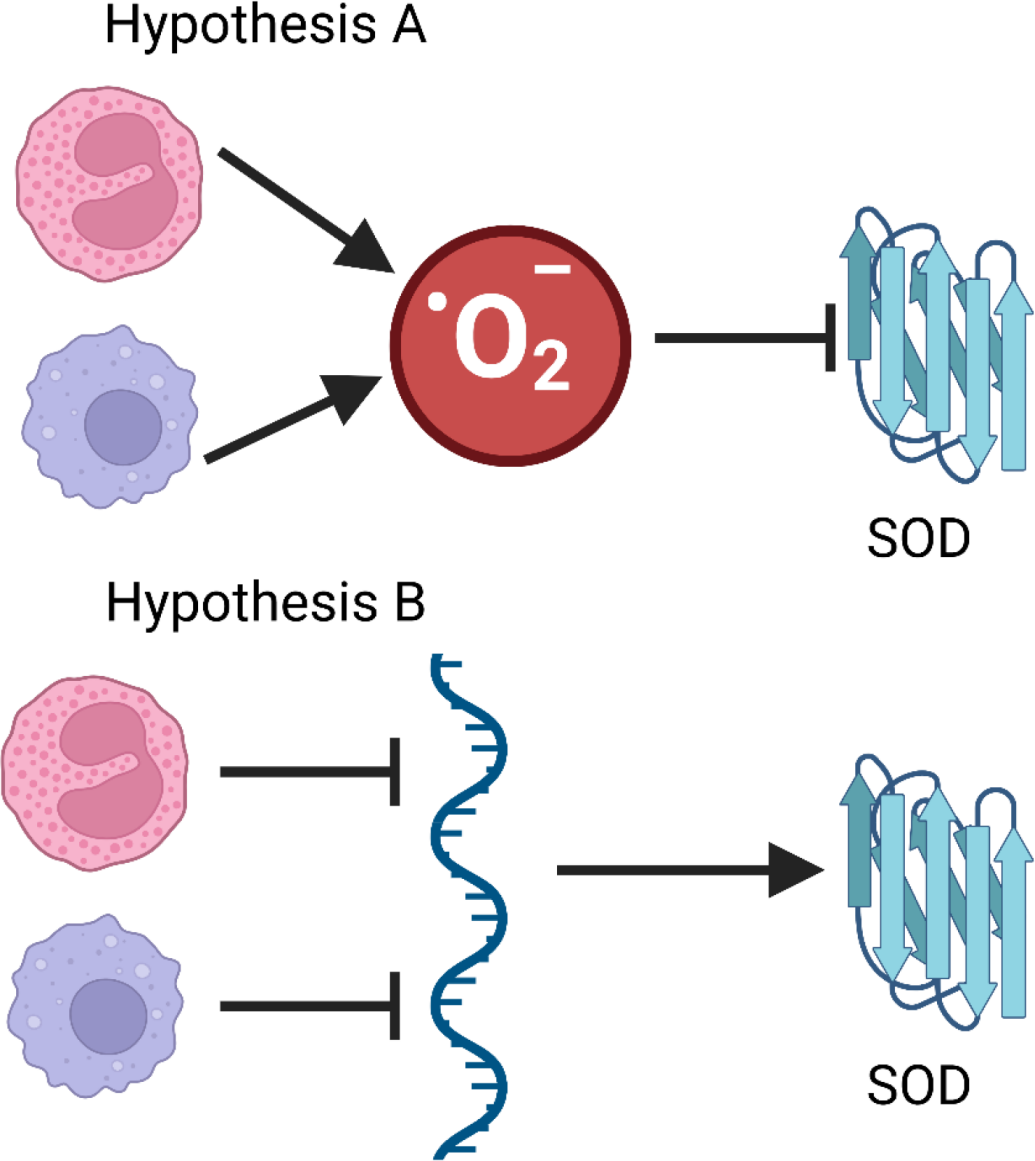
Proposed mechanisms of SOD activity and expression inhibition in eosinophilic sinusitis.

SOD is a class of enzymes that are part of body’s antioxidant defense system. These enzymes help counteract cellular oxidative stress, lipid metabolism, inflammation, and oxidation. As per Zheng et al. (38), SOD exists in three isoforms: intracellular copper-zine SOD (Cu,Zn-SOD), mitochondrial manganese SOD (Mn-SOD), and extracellular SOD(EC-SOD). SOD has been widely studied and applied in clinical settings. Therefore, in the present study, the focus was on studying SOD as an antioxidant. Ono et al. (16) examined the relationship between eosinophil infiltration, epithelial damage, and SOD deficiency in ECRS and obtained the following results: (1) SOD activity in the non-ECRS group, the CRS group with a low eosinophil count of less than 100/microscopic field (400× magnification), was significantly lower compared with that in the control group with normal mucosal membranes;(2) immunostaining for both CuZn-SOD and Mn-SOD was significantly lower in the ECRS group, the CRS group with a high eosinophil count of more than 350/microscopic field (400× magnification), compared with the non-ECRS and control groups; (3) CuZn-SOD mRNA levels were significantly reduced in the ECRS group compared with the control group; (4) MN-SOD mRNA levels were significantly lower in the ECRS group compared with the non-ECRS and control groups; (5) the degree of epithelial damage and CT scores inversely correlated with CuZn-SOD and Mn-SOD immunoreactivity; (6) the mean percentage of epithelial cells exhibiting Cu,Zn-SOD and Mn-SOD positive reactions in the non-ECRS and control groups was significantly greater than in the ECRS group. (7) Cu,Zn-SOD and Mn-SOD mRNA levels were significantly lower in the ECRS group compared with the control group. These findings suggest two hypotheses, described in **Figure 1**: (A) damage-inducing oxidants from eosinophils and macrophages may deplete SOD in the epithelia of ECRS, and (B) some cytotoxic factors secreted from eosinophils and macrophages may suppress the production of SOD and finally overwhelm the cytoprotection derived from epithelial cells in ECRS.

Nishi et al. (12) reported that SOD overexpression could reduce the expression of monocyte chemotactic protein-1 (MCP-1), which can attract macrophages, after transient focal cerebral ischemia in mice. Thus, the epithelial damage characteristically observed in ECRS is thought to be brought about by interactions between cytotoxic enzymes or cytokines and cytoprotective factors such as SOD. Ono et al. (16) further supports the hypothesis that a loss of Cu, Zn-SOD in the epithelial cells of ECRS leads to an increase in IL-17A, macrophages, and MUC5AC, contributing to macrophage infiltration and mucus overproduction (**Table 4**).

**Table 4:** Inhibitors of eosinophilic sinusitis (ECRS) pathology.

Comparison/Measurement	Finding/Relationship	Statistical Significance (P-value)
Percentage of HO-1 + cells	ECRS < Non-ECRS	P < 0.001
Percentage of Cu,Zn-SOD + cells	ECRS < Non-ECRS	P < 0.01
Cu,Zn-SOD+ cells vs. CD68+ (macrophage) cells	Negative correlation	P < 0.01
Cu,Zn-SOD+ cells vs. IL-17A+ cells	Negative correlation	P < 0.05
Cu,Zn-SOD+ cells vs. MUC5AC+ cells	Negative correlation	P < 0.05

(1) + means the cell is expressing a significant amount of the protein/marker in question. (2) The data suggests that ECRS patients show significantly lower levels of antioxidant markers (HO-1 and Cu,Zn-SOD) compared to non-ECRS patients, with various negative correlations involving inflammatory markers.

Even in asthma which has a high coexistence rate with ECRS, Pae et al. (39) reported that the upregulation of antioxidants significantly mitigated airway inflammation, mucus hypersecretion, and hyper-responsiveness in a model of allergic asthma. Haube et al. (40) also demonstrated that antioxidants effectively reduced bacteria-induced mucin mRNA expression (MUC5AC) in lumen airway mucosa. As per Ono et al. (16), epithelial damage shows a significant inverse correlation with the percentage of Cu, Zn-SOD-positive epithelial cells.

Here, we will discuss SOD from a molecular perspective.

Tainer et al. (41) described how superoxide radicals, toxic molecules responsible for oxidative stress, are converted into hydrogen peroxide (H_2_O_2_) by SOD to mitigate oxidative stress damage. H_2_O_2_ is less reactive than superoxide radicals and can be broken down into water and oxygen by catalase.

Fita et al. (42) have proposed a histidine-dependent breakdown mechanism of H_2_O_2_ by catalase, and Kato et al. (43) have proposed a second histidine-dependent mechanism, as well as a histidine-independent breakdown of H_2_O_2_. As per Goyal et al. (44), the active site of catalase is made up of iron (III) and a porphyrin ring. As per Pan et al. (45), iron is oxidized by H_2_O_2_ to form water and oxoferryl (V) porphyrin. However, because oxoferryl (V) porphyrin is unstable, the iron takes an electron from the porphyrin ring, leaving it with an unpaired electron. This creates the oxoferryl (IV) porphyrin radical cation. As per Putnam et al. (46), this cation is stabilized by electron donation from an ionized tyrosine amino acid residue (Tyr358 in humans), which reduces the electron deficiency of the cation. As per Goyal et al. (44), this radical is reduced back to iron (III) by another H_2_O_2_ molecule, which is broken down into water and oxygen.

SOD is known to have a short half-life regardless of its type. Joseph (47) reported that the half-life of SOD varies greatly, ranging from only 100 hours in kidney cells to over a year when undergoing axonal transport in an environment with few proteasomes in the axon.

**Figure 2:**
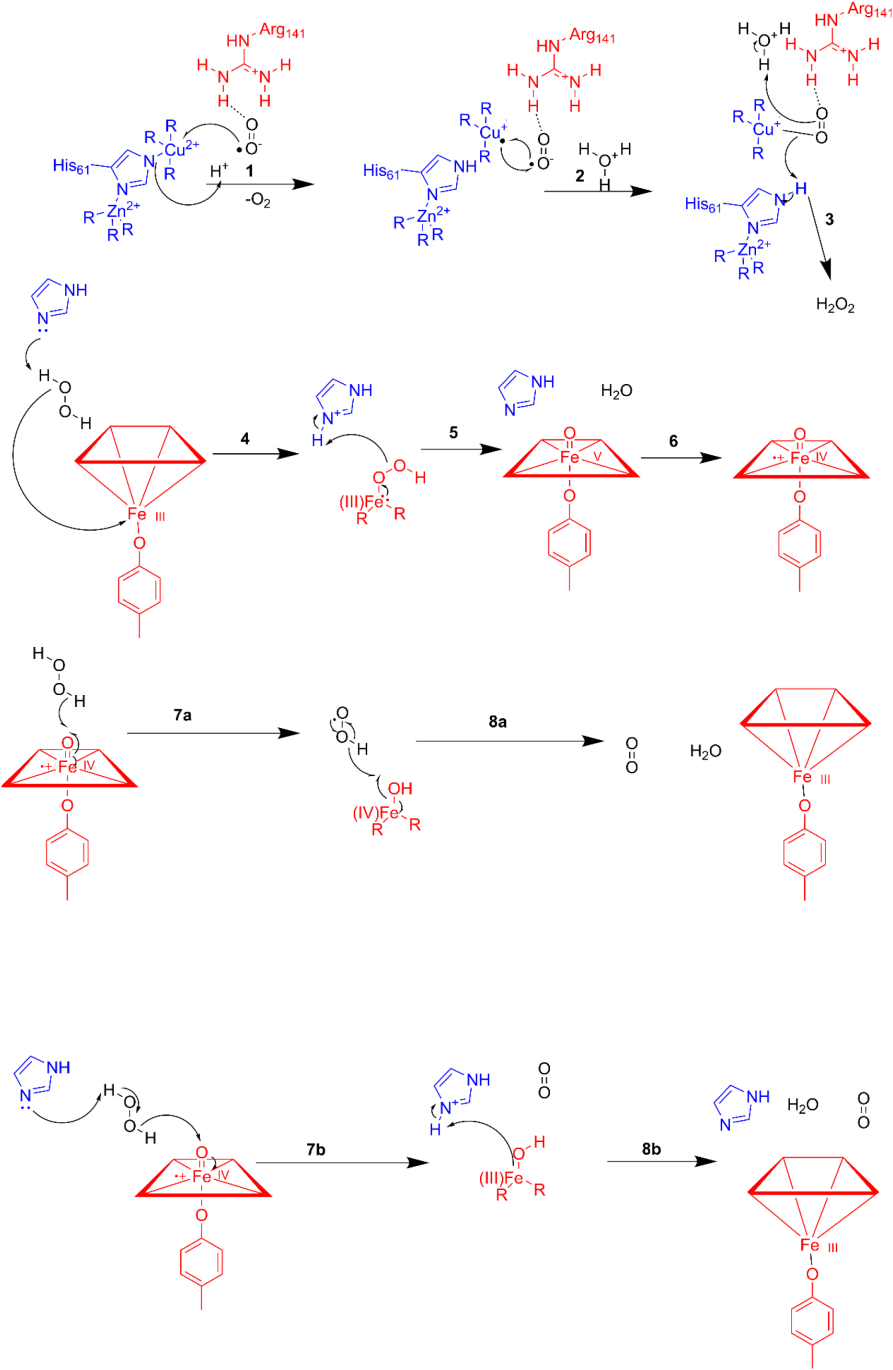
Electron delocalization chemical flowsheet of the mechanism by which superoxide radicals are converted into H_2_O_2_ and subsequently broken down into water and oxygen.

Based on this critical analysis of the aforementioned scientific literature, this study has developed a chemical flowsheet based on electron delocalization, shown in **Figure 2**. This chemical flowsheet demonstrates the mechanism of superoxide radical conversion into H_2_O_2_ and the subsequent breakdown of H_2_O_2_ into water and oxygen.

In **Figure 2**, steps 1–3 summarize the antioxidant function of superoxide dismutase (SOD), which converts superoxide radicals into hydrogen peroxide (H_2_O_2_) through copper redox cycling. Steps 4–8 illustrate the subsequent detoxification of H_2_O_2_ by catalase within its heme-containing active site, where iron undergoes transient oxidation to form a high-valent oxoferryl intermediate before being reduced back to its resting state, ultimately generating water and oxygen. Two mechanistic routes—histidine-dependent and histidine-independent—have been described for this catalytic turnover.

For the purposes of ECRS pathophysiology, the key concept is that efficient cycling of SOD and catalase prevents accumulation of reactive oxygen species (ROS). Impairment or overwhelming of these enzymatic pathways may permit excessive H_2_O_2_ and downstream oxidative signaling, thereby contributing to epithelial damage, macrophage recruitment, cytokine amplification (e.g., IL-17A), and mucus overproduction. Thus, **Figure 2** is intended not as a detailed enzymological reconstruction, but as a conceptual framework linking antioxidant enzyme kinetics to oxidative stress–driven inflammation in ECRS.

Based on the above, it was concluded that the therapeutic targets for oxidative stress in ECRS are the following:

- the homologs of Asn 141 and His 61 in humans for SOD
- the ferrylporphyrin (IV) radical cation for catalase
- Tyr358 for catalase

Regarding the individual catalytic mechanisms of SOD and catalase, prior studies have presented these mechanisms in isolation from disease-specific inflammatory contexts. To our knowledge, this is the first time that the electron delocalization steps of SOD and catalase have been reconstructed into a unified chemical flowsheet and explicitly connected to the clinical pathophysiology of ECRS. Previous literature describes SOD reduction–oxidation chemistry and catalase intermediates independently; however, no prior publication has integrated (1) Cu(II)/Cu(I) cycling of SOD, (2) oxoferryl (IV) porphyrin radical stabilization, and (3) histidine-dependent and -independent catalase pathways into a single disease-oriented oxidative stress network. Furthermore, this is the first model that directly connects these detailed redox mechanisms with downstream inflammatory mediators relevant to ECRS (IL-17A, MCP-1, macrophage recruitment, MUC5AC), thereby transforming classical enzymology into a mechanistic disease framework. This integration represents a conceptual advance from isolated enzyme kinetics toward a systems-based oxidative stress model specific to ECRS.

## 4 The role of HO-1 in ECRS

HO is an enzyme with multiple functions, capable of catabolizing heme to produce carbon monoxide, free iron, and biliverdin. As per Ponaka et al. (48), biliverdin is rapidly converted to the antioxidant bilirubin by biliverdin reductase, and free iron is sequestered by ferritin. Three isoforms of HO have been identified. According to Choi et al. (49), HO-1, a 32-kDa protein, can be induced in cells by various stimuli, including oxidative stress, heavy metals, ultraviolet light, as well as heme and its derivatives. Horvath et al. (50) reported that chronic inflammatory lung diseases are associated with increased production of oxidants, and the induction of HO-1 by ROS serves as a cytoprotective mechanism against oxidative stress.

Elhini et al. (51) reported upregulation of HO-1 in the nasal mucosa of patients with allergic rhinitis. In Kawano et al. (52), HO-1 expression in the mucosal epithelium of chronic rhinitis without eosinophilic infiltration was significantly enhanced compared to chronic rhinitis with eosinophilic infiltration. Conversely, as per Kawano et al. (52), the number of macrophages expressing HO-1 was significantly higher in CRS with eosinophilic infiltration than in CRS without eosinophilic infiltration.

It was observed that the number of macrophages expressing HO-1 was significantly greater in ECRS than in CRS without eosinophilic infiltration. It was considered that both macrophages and epithelial cells produce HO-1 to protect themselves against oxidants generated by macrophages, which are predominant in tissues of ECRS. Our previous research suggests that a reduction in the production of HO-1 in epithelial cells and an enhanced macrophage infiltration are associated with epithelial damage in ECRS. Moreover, the epithelial damage observed in ECRS is thought to be driven by interactions between cytotoxic enzymes or cytokines and cytoprotective factors, such as HO-1. Here, we have examined HO-1 in detail from a molecular perspective. One of the products of the degradation heme, the oxygen-binding protein, by heme oxygenase (HO-1) is carbon monoxide (CO). Otterbein et al. (53) investigated the impact of sublethal doses of CO on inflammation in mice. According to them (53), CO inhibited inflammation by preventing macrophages from releasing the following proinflammatory cytokines in mice who come into contact with lipopolysaccharide (LPS): (a) tumor necrosis factor-alpha (TNFα), (b) interleukin-6 (IL-6), (c) interleukin 1-beta (IL-1β), and (d) macrophage inflammatory protein-1beta (MIP-1β). LPS originates from gram-negative bacteria. According to Ryter et al. (54), LPS is a component of the bacterial cell wall in Gram-negative bacteria. According to Hambleton et al. (55) and Raingeaud et al. (56), LPS triggers inflammation by activating MAP kinases, such as c-Jun N-terminal kinases and p38, respectively. As per Otterbein et al. (57), CO also inhibits the production of inflammatory cytokines TNFα, IL-1β, and interleukin-6 (IL-6) caused by oxidative stress. Otterbein et al. (53, 57) state that the anti-inflammatory effect of CO is due to its selective activation of the p38 and the MKK3, which are both MAP kinases. This anti-inflammatory effect may seem counterintuitive because LPS also triggers inflammation by activating p38. However, Otterbein et al. (53) state that CO causes hyperstimulation of p38, which is inhibitory to inflammatory cytokine production, in contrast to the modest p38 activation triggered by LPS.

As per Toribio et al. (58), T lymphocytes, cells which play a crucial part in the immune response, produce interleukin 2. Toribio et al. (58) state that T cells proliferate after interleukin 2 (IL-2) binds to the interleukin 2 receptor (IL-2R). Desilva et al. (59) confirmed that the inhibition of the extracellular signal-regulated kinase (ERK) decreased the amount of IL-2 mRNA, thereby blocking T cell proliferation due to a lack of IL-2 production. Pae et al. (60) demonstrated that CO prevents T cell proliferation by suppressing IL-2 secretion, most likely by inhibiting the ERK kinase. ERK is inhibited by preventing its phosphorylation by the mitogen-activated protein kinase kinase (MEK). CO also inhibited inflammation by reducing the production of proinflammatory prostaglandins. As shown in **Table 3** of Chandrasekharan et al. (61), cyclooxygenase enzymes produce prostaglandins, which, according to Yin et al. (62), are a family of lipid compounds derived from arachidonic acid, the substrate of cyclooxygenase. Williams et al. (63) found that prostaglandins are responsible for vasodilatation, which increases the production of plasma exudation (leaking) into the inflammatory site after increased vascular permeability. According to Rodrigues Soares et al. (64), this process is essential for the recruitment of white blood cells to the inflammatory site. As per Wang et al. (65), the production of the proinflammatory cytokines like IL-6 is dependent on the binding of the PGE2 prostaglandin to the prostaglandin E2 receptor (PE2). Suh et al. (66) demonstrated that CO decreased macrophage secretion of prostaglandins by reducing the expression of CCAAT/enhancer-binding protein δ (C/EBP δ), a protein which, as per Chen et al. (67) is responsible for the synthesis of the prostaglandin-producing cyclooxygenase 2. The decrease in C/EBP δ production leads to a reduction of COX-2 synthesis. This is important because, as previously mentioned by Chandrasekharan et al. (61), COX-2 causes prostaglandin production. Also, according to Ejima et al. (68), COX-2-deficient mice are resistant to inflammation and death caused by infection.

**Figure 3:**
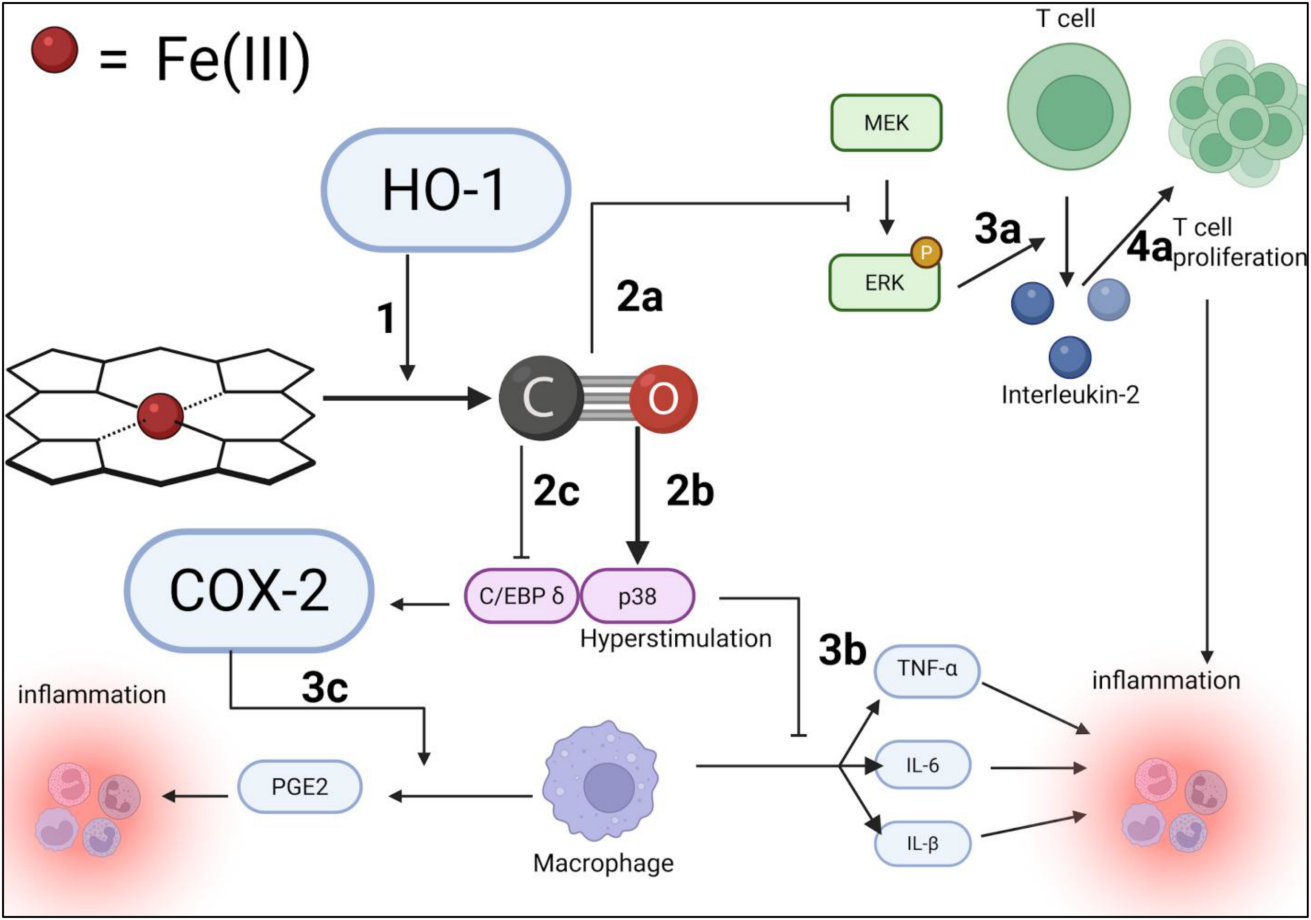
Biochemical flowsheet of the unified CO-based signaling pathway of inflammation inhibition mechanisms.

Based on this critical analysis of the aforementioned scientific literature, a unified signaling pathway including CO and all major inflammatory suppression mechanisms is shown in the biochemical flowchart in **Figure 3**. This is probably the first explanation of this phenomenon.

Step 1 shows the production of CO by HO-1 from heme. Step 2 summarizes the three distinct pathways (a, b and c) by which CO inhibits inflammation. Step 2a illustrates the inhibition of ERK phosphorylation activation by CO. Step 3a demonstrates the induction of IL-2 production in T cells by phosphorylated ERK. Step 4A shows the proinflammatory induction of T cell proliferation by IL-2. Step 2b shows the hyperstimulation of the p38 kinase by CO. Step 3b shows the inhibition of the macrophage production of proinflammatory cytokines TNF-α, IL-6 and IL-β. Step 2c shows the inhibition of the production of C/EBP δ by CO. Step 3c shows the increase in expression of COX-2 by C/EBP δ. Step 4c shows the production of prostaglandins by COX-2, causing inflammation.

Altogether, the following therapeutic targets to combat inflammation for ECRS can be identified.

- MKK3
- MEK
- IL-2R
- ERK
- C/EBPs
- COX-2
- Prostaglandins
- prostaglandin receptors

Regarding anti-inflammatory effects of carbon monoxide and HO-1 signaling through p38, ERK, or C/EBPδ pathways, these mechanisms have not previously been synthesized into a unified signaling framework within the context of ECRS. Existing publications describe CO-mediated modulation of individual pathways (e.g., ERK inhibition, p38 hyperactivation, or COX-2 suppression) separately and largely outside sinus inflammatory disease models. **Figure 3** represents the first comprehensive integration of these three major CO-dependent anti-inflammatory axes into a single pathway network that links T-cell proliferation, macrophage cytokine production, prostaglandin synthesis, and MAPK modulation under one mechanistic framework. Importantly, this model positions HO-1-derived CO not merely as an antioxidant byproduct but as a central regulatory node coordinating multiple inflammatory suppression mechanisms relevant to ECRS pathology. This systems-level reconstruction extends beyond prior fragmented reports and provides the first disease-focused integration of HO-1/CO signaling in eosinophilic sinus inflammation.

## 5 Other important signaling pathways involved in ECRS: HIF-1, PTPN2, B cells, and the inflammasome

Multiple other signaling and biochemical pathways are involved in ECRS. As per Wang et al. (69), the hypoxia-inducible factor 1 (HIF-1) is activated in response to hypoxia. It increases the synthesis of erythropoietin, the protein responsible for increasing erythropoiesis (the production of red blood cells). O’Rourke et al. (70) found that HIF-1 regulates the production of other proteins as well. As per Chien et al. (71), HIF-1 expression is higher in pathological nasal polyps than in healthy epithelial nasal tissue. According to Otani et al. (72), tight junctions are essential for the proper functioning of epithelial tissue. As per Sugita et al. (73), tight junction dysfunction causes an increased pathological flux through the epithelial membrane due to increased permeability, as well as immune cell activation and chronic inflammation. Zhang et al. (74) discovered that increased expression of HIF-1 in nasal tissue inhibited the transcription of protein tyrosine phosphatase non-receptor type 2 (PTPN2). As per Zhang et al. (74), this decreased the expression of three proteins necessary for forming tight junctions: claudin-4, occludin and ZO-1 (zonnula occludens 1). This leads to a breakdown of epithelial junctions, causing chronic inflammation in ECRS.

Based on the current critical analysis of the aforementioned scientific literature, it was concluded that these findings demonstrate two additional therapeutic targets in ECRS:

- HIF-1
- PTPN2.

Moreover, as per Laidlaw et al. (75), eosinophiles are not the main cause of chronic inflammation in ECRS with nasal polyps. Laidlaw et al. depleted 97% of eosinophiles in nasal polyps of CRS patients and found no significant reduction in nasal polyp swelling or improvement in CRS symptoms. On the contrary, though eosinophiles may play a crucial role in the initial stages of ECRS chronic inflammation, B cells are increasingly seen as the main cells behind CRS pathogenesis. As per Buchheit et al. (76), B cells in airway tissue have IL-5 receptors, and IL-5 is one of the key interleukins produced by Th2 cells responsible for the type 2 inflammatory response in CRS as was mentioned in section 2 of this article. As per Schwitzguébel et al. (77) and Tan et al. (78), immunoglobulin (antibody) deficiency in IgG1-3D, IgA and IgM antibodies as well as autoreactive IgG and IgA antibodies, respectively, all produced by B cells, were proven to be linked to CRS. Most importantly, the pathological overproduction of IgE, an antibody, which, as per Gould et al. (79), is responsible for sensitizing immune cells to toxic or foreign substances known as allergens, has been shown to play an important role in the activation of immune cells and subsequent secretion of inflammatory cytokines. Buchheit et al. (76) demonstrated an increase in IgE production in nasal polyps. Zhang et al. (80) also demonstrated that IgE-producing B cells contribute to the chronic inflammation by continuously activating mast cells. Mast cells, as per Moon et al. (81), release granules containing lysosomal proteins such as acid hydroxylases that degrade cells, and histamine, which, as per Lopez (82) causes vasodilatation. As per Gevaert et al. (83), patients with severe CRS treated with the drug Omalizumab reported significant improvement in symptoms and quality of life. According to Kaplan et al. (84), Omalizumab is an antibody drug that selectively binds to IgE and prevents it from activating mast cells.

The inflammasome is also involved in the development of CRS due to the implication of IL-17A in the disease. As per Li et al. (85), in CRS, the binding of IL-17A to the IL-17A receptor promotes the phosphorylation and activation of the ERK protein. As per Ghonime et al. (86), phosphorylated ERK is necessary for the proper interaction between the Apoptosis-associated speck-like protein containing a caspase recruitment domain (ASC) and nucleotide-binding oligomerization domain-like receptor proteins (NLRP) such as NLRP3. Compan et al. (87) shows that ASC is necessary for bridging between NLRP3 and procaspase-1, which results in the formation of caspase-1. Bai et al. (88) reported that NLRP3 forms the NLRP3 inflammasome after detecting irritants and/or microbes, and causes pyroptosis, a type of cell death that releases the pro-inflammatory cytokines IL-1β and IL-18 outside the cell. According to Li et al. (85), the NLRP3 inflammasome causes pyroptosis by activating caspase-1, which cleaves and activates gasdermin-D (GSDMD), IL-1β and IL-18. As mentioned by Bai et al. (88), GSDMD is a protein that causes cell death by creating pores in the cell membrane, which also permits the release of the inflammatory cytokines. Li et al. (85) determined that IL-17A contributes to pyroptosis-induced inflammation in CRS with nasal polyps and increases patient resistance to steroid treatment, which, as per Head et al. (89), is a recommended and effective treatment in reducing CRS symptoms. Also, Lin et al. (90) demonstrated that both NLRP3 and caspase-1 were overexpressed specifically in ECRS.

Altogether, the current critical analysis of these aforementioned publications demonstrates that the following proteins are considered to be important potential therapeutic targets for ECRS:

- NLRP3
- ERK
- MEK
- IgE

**Figure 4:**
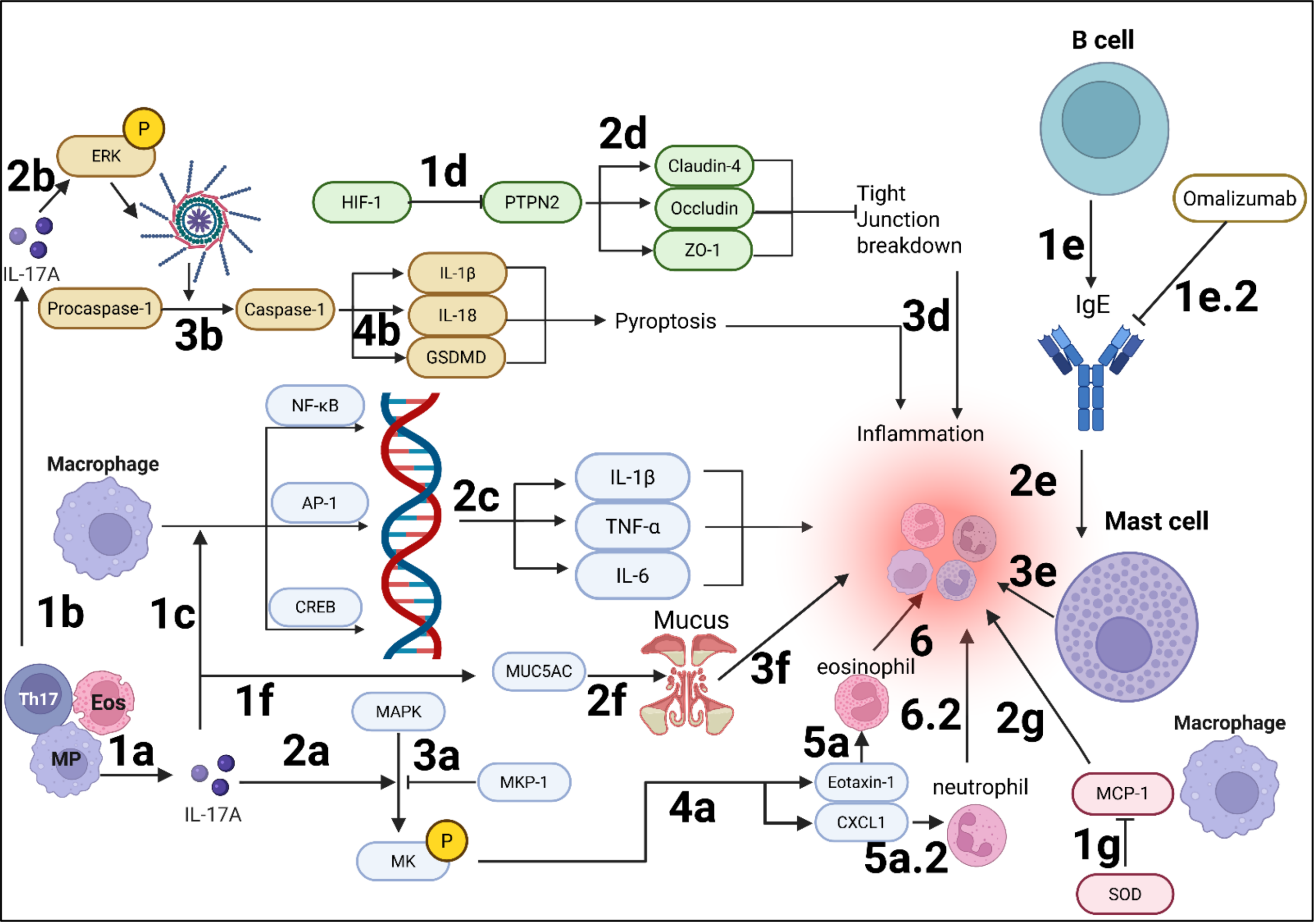
Biochemical flowsheet of the unified signaling pathway of inflammation.

Based on this critical analysis, this study developed a biochemical flowsheet of a unified signaling pathway involved in the inflammatory processes described in this paper. This biochemical flowsheet is shown in **Figure 4**.

In **Figure 4**, steps 1a and 1b are the two different inflammatory pathways following the production of IL-17A by T_H_17 cells. Step 2a is the induction of MK phosphorylation by MAPK caused by IL-17A. Step 3a is the dephosphorylation and subsequent inhibition of MK by MKP-1. Step 4a is the induction of eotaxin-1 and CXCL1 production by phosphorylated MK. Steps 5a and 5.2a are the attraction of eosinophiles and neutrophiles to the inflammatory site by eotaxin-1 and CXCL1, respectively. Steps 6 and 6.2 are the inflammation caused by eosinophiles and neutrophiles. Step 2b is the induction of ERK phosphorylation by IL-17A. Step 3b is the induction of procaspase-1 cleavage by ERK. Step 4b is the cleavage and activation of IL-1β, IL-18 and GSDMD by caspase-1, causing pyroptosis (inflammatory cell death). Step 1c is the induction by IL-17A of NF-kB, AP-1 and CREB transcription factor accumulation to DNA and enhancer/promoter sequences. Step 2c is the induction of transcription and translation of proinflammatory cytokines IL-1β, TNF-α, IL-6 by the transcription factors NF-kB, AP-1 and CREB. Step 1d is PTPN2 expression inhibition by HIF-1. Step 2d is the increase in the production of proteins claudin-4, occludin and ZO-1, which prevent tight junction breakdown and inflammation. Step 1e is the production of IgE antibodies by B cells. Step 1e.2 is the inhibition of IgE antibody activity by the drug Omalizumab. Step 2e is the activation of mast cell degranulation by IgE antibodies. Step 3e is the subsequent inflammation caused by mast cell degranulation. Step 1f is the increase in MUC5AC mucin protein production caused by IL-17A. Step 2f is the increase in mucus secretion caused by MUC5AC. Step 3f is the increase in inflammation caused by mucus hypersecretion. Step 1g is the inhibition of MCP-1 protein production by SOD. Step 2g is the inflammation caused by MCP-1, which recruits the macrophage to the inflammatory site.

IL-17A signaling, HIF-1 regulation, inflammasome activation, tight junction disruption, and IgE-mediated mast cell activation have not previously been integrated into a single unified inflammatory network specific to ECRS. Existing studies typically examine these pathways independently within asthma, autoimmune disease, oncology, or general CRS contexts. To our knowledge, **Figure 4** is the first model to interconnect: (1) IL-17A-driven MAPK activation, (2) ERK-dependent inflammasome signaling and pyroptosis, (3) HIF-1–mediated tight junction disruption via PTPN2 suppression, and (4) B cell/IgE amplification of mast cell inflammation, into one coordinated molecular framework. By mapping these previously compartmentalized pathways into a single flowchart, this study moves beyond endotype classification and proposes a mechanistic convergence model explaining how type 1, type 2, and type 3 inflammatory axes interact in ECRS. This integrative representation provides a novel systems-level understanding of ECRS pathophysiology and allows identification of therapeutic targets across oxidative, immunologic, and barrier-regulatory domains simultaneously — an approach not previously articulated in the literature.

## 6 Practical guidance regarding therapeutic targets and their stratification

This paper identifies several therapeutic targets for treating ECRS. In this section we will establish which targets have drugs used against them in other diseases and how they can be used in the context of ECRS. Therefore, in this section, we identify for the first time how drugs used in other diseases can be used to treat ECRS.

First, we have the HIF protein. This protein already has a drug related to it called roxadustat (91). This drug inhibits the HIF prolyl hydroxylase domain enzymes (PHD enzymes) which cause the degradation of the HIF protein (identified target 1 for ECRS in this study). Roxadustat therefore increases HIF protein quantity. Other PHD inhibitors exist, such as Daprodustat (92), Vadadustat (93), Desidustat (94), Enarodustat (95), and Molidustat (96). A HIF inhibitor by the name of Belzutifan also exists (97). Our flowsheet in **Figure 4** indicates that HIF counteracts the anti-inflammatory effects mediated by PTPN2 (Identified target 2). Therefore, in the context of ECRS, we propose evaluating whether treatment with belzutifan could attenuate disease severity by inhibiting HIF.

PTPN2 inhibitors, such as ABBV-CLS-484, also exist (98). However, as per **Figure 4**, in the context of ECRS, it is in the patient’s best interest to use PTPN2 activators such as spermidine to stem ECRS-induced inflammation (99). Quercetin can also increase Claudin-4 expression (identified target 3), which could help in reducing ECRS induced inflammation (100), and rapamycin, the inhibitor of the mechanistic target of rapamycin (mTOR) could also reduce ECRS inflammation by increasing the expression of occludin and ZO-1 (Identified targets 4 and 5) (101).

Our flowsheet in **Figure 4** also established that NLRP3 (Identified target 6), by forming the inflammasome, increases inflammation in ECRS. Therefore, an inflammasome inhibitor which targets NLRP3 such as MCC950 (102) could reduce ECRS-induced inflammation. As previously covered, omalizumab targets and inhibits IgE to combat ECRS induced inflammation (Identified Target 7).

Our flowsheet in **Figure 3** established that CO (Identified target 8) reduces inflammation and, therefore, the appropriate course of action in the case of ECRS-induced chronic inflammation would be to increase CO safely through carbon monoxide-releasing molecules (CORMs) (103). Regarding COX-2 specifically (Identified Target 9), it is in the ECRS patient’s best interest to inhibit COX-2, which also results in the inhibition of PGE 2 (Identified Target 10). This is possible by using celecoxib, a drug used to treat back pain (104), just like paracetamol. The latter, however, has no significant inhibitory effect on inflammation (105). Although Ibuprofen has been shown to help CRS patients manage pain better following surgery (106), it was not found to stem the underlying cause of CRS. This may be because ibuprofen is a non-selective inhibitor of both Cox 1 and 2 (107). Cox-1 helps preserve homeostasis, whereas Cox-2 is induced in response to inflammation (108). Therefore, we believe Ibuprofen’s inhibition of Cox 1 may actually worsen ECRS symptoms in the long term, and we propose for the first time a COX-2 specific inhibitor such as celecoxib as an optimal novel treatment for ECRS. P38 (Identified Target 11), on the contrary, as per our **Figure 3** flowsheet, should be hyperstimulated to inhibit inflammation, and this is possible by using sodium salicylate (109). C/EBP δ, which must be inhibited to reduce inflammation in ECRS, as per **Figure 3**, can be inhibited via the natural products rosmanol and inotilone (110).

The key targets to inhibit regarding pyroptosis are GSDMD (Identified Target 13), IL-18 (Identified Target 14) and IL-1β (Identified Target 15). The activation of all three is dependent on caspase 1 and its critical catalytic cysteine, C285. This cysteine can be inhibited reversibly by Pralnacasan or VX-765, and irreversibly by IDN-6556 (111).

We strongly believe that the anti-interleukin 2 receptor (the receptor of Identified Target 16) antibodies basiliximab and daclizumab (112) could be beneficial in inhibiting the proliferation of T cells which occurs in ECRS and contributes to the chronic inflammation.

Regarding MEK (Identified Target 17), which promotes T cell proliferation, MEK inhibitors have been shown to inhibit the growth of melanoma cells, induce tumor cell death, and tumors have been shown to take longer to regrow when MEK inhibitors are used in combination therapies (113). We propose using MEK inhibitors in combination with basiliximab and daclizumab in the context of ECRS where T cell proliferation occurs in order to reduce inflammation and block the interleukin-2 producing pathway at different levels to ensure optimal inhibition. We also postulate that combination therapy using MEK inhibitors to stem T cell proliferation can also be used along with ERK (Identified Target 18) inhibitors such as SKLB-D18 and BVD-523 (114).

With regards to oxidative stress, we strongly believe that ECRS inflammation can be mitigated with the therapeutic application of curcumin (115). This compound has been shown to increase the activity of the antioxidant enzyme catalase (Identified Target 19). Regarding oxoferryl (IV) porphyrin radical cation, otherwise known as compound I (Identified Target 20), we inferred from previous literature (116) that, at the conceptual level, catalase redox potential could be increased by positioning a nearby positively charged molecule similar to 2-N-methylpyridinium to enhance compound I reactivity, and our team is currently working on this. Both these methods are direct ways of inhibiting hydrogen peroxide (Identified Target 21), which in turn should decrease oxidative stress and therefore inflammation in ECRS.

Eotaxin-1 (Identified Target 22) and its ability to recruit eosinophils to the inflammation site can also be inhibited by using the monoclonal antibody bertilimumab (117), which we propose as a potential therapy specifically for treatment in the early stages of the disease. Regarding oxidative stress, we propose that, in the context of ECRS, our supplement twendee X would be a right choice as an antioxidant. Twendee X has been proven to have a strong antioxidant and healing effects in other diseases (118–136).

Regarding the three proinflammatory transcription factors, numerous therapeutic inhibitors such as the NF-κB (Identified Target 23) inhibitor Emetine (137) exist, which we believe would be useful in reducing chronic inflammation in ECRS. We believe AP-1 (Identified Target 24) therapeutic drug inhibitors such as T-5224 (138) would also be useful in treating ECRS inflammation. Likewise, for the possible CREB-induced inflammation (Identified Target 25) in ECRS, we believe that CREB gene transcription inhibitors such as 666–15 (139) would be optimal as a treatment. Inhibiting NF-κB, AP-1 or CREB would lead to the inhibition of IL-6 (Identified Target 26) and TNF-α (Identified Target 27).

## 7 Clinical implications of the unified redox-inflammatory model

The molecular mechanisms described in this study provide a direct framework for understanding the clinical hallmarks of Eosinophilic Chronic Rhinosinusitis (ECRS), including persistent obstruction, anosmia, and high surgical recurrence. Rather than viewing these as isolated symptoms, our model suggests they are the measurable outcomes of a unified redox-inflammatory circuit. For instance, the observed increase in CD68-positive macrophage infiltration does not merely serve as a histopathological marker; it correlates significantly with basement membrane thickening, higher CT scores, and the severity of comorbid asthma. These macrophages drive a feed-forward loop of IL-17A production and oxidative stress, which clinically manifests as increased mucosal edema and a greater nasal polyp burden.

Furthermore, the central role of IL-17A facilitates a transition from molecular signaling to clinical phenotype by inducing MUC5AC expression and amplifying MAPK signaling. This explains the characteristic viscous mucus hypersecretion and the resulting anosmia seen in patients. Crucially, the activation of the NLRP3 inflammasome by IL-17A has been linked to corticosteroid resistance. Therefore, the signaling components of our model—specifically MAPKs, ERK, and caspase-1—may serve as vital biomarkers to predict a patient’s responsiveness to conventional steroid therapy, allowing for earlier stratification toward alternative treatments.

## 8 Molecular integration of antioxidants and the cytokine milieu

A key finding of this integrated mapping is that oxidative stress and cytokine signaling are mutually amplifying systems. Antioxidant enzymes such as SOD, catalase, and HO-1 function as “molecular gatekeepers” that control the intensity and duration of the inflammatory response. In the ECRS microenvironment, a deficiency in Cu,Zn-SOD leads to the accumulation of superoxide anions, which promotes the nuclear translocation of NF-κB and the subsequent transcription of pro-inflammatory cytokines like IL-1β and TNF-α. This deficiency actively drives macrophage recruitment and sustains their activation, creating a self-perpetuating circuit of tissue damage.

Similarly, the coordination between SOD and catalase is essential for regulating hydrogen peroxide (H_2_O_2_), a diffusible signaling molecule that prolongs MAPK phosphorylation. When this redox balance is disrupted, the resulting H_2_O_2_ accumulation potentiates IL-17A-driven eosinophil recruitment and barrier dysfunction. Additionally, the HO-1/CO axis provides an extra layer of regulatory control; HO-1-derived carbon monoxide can suppress ERK-dependent T-cell proliferation and COX-2-mediated prostaglandin production. Together, these interactions provide a mechanistic justification for targeting redox pathways not simply to neutralize free radicals, but to modulate the very intracellular cascades that govern cytokine production.

## 9 Therapeutic transitions: from systemic risks to topical precision

While identifying these signaling networks reveals numerous therapeutic targets, the systemic modulation of redox-sensitive pathways carries inherent risks, such as immunosuppression or off-target kinase inhibition. Given that ECRS pathology is anatomically localized to the sinonasal mucosa and ethmoid sinuses, it is uniquely suited for targeted intranasal interventions. Localized delivery of antioxidant-enhancing compounds—such as SOD mimetics, catalase-enhancing agents, or nanoparticle-based stabilizers—offers a compelling strategy to restore epithelial redox balance while preserving systemic immune homeostasis.

Topical administration allows for higher drug concentrations at the target tissue with reduced systemic absorption, potentially mitigating the toxicity associated with systemic CO-releasing molecules or kinase inhibitors. These localized approaches could effectively reduce steroid dependence, decrease postoperative recurrence, and lower the requirements for expensive biologic therapies. By stabilizing the epithelial barrier through the targeted restoration of tight junction proteins like claudin-4 and ZO-1, these formulations may stop chronic inflammatory amplification at its source. Ultimately, integrating localized antioxidant therapy with existing treatments represents a promising path toward precision medicine in ECRS management.

## 10 Conclusion

Previous studies have clearly shown significant negative correlations between an antioxidant Cu, Zn-SOD and key exacerbating factors of ECRS, including: CD68, IL-17A, and MUC5AC. The loss of Cu,Zn-SOD in the epithelial cells of ECRS likely leads to an increase in IL-17A, macrophage infiltration, and MUC5AC expression, resulting in enhanced mucus production and further macrophage infiltration. Moreover, both macrophages and epithelial cells generate HO-1 as a defense mechanism against oxidants produced by macrophages, and a reduction of HO-1 production in the epithelium, as well as macrophage infiltration, causes epithelial damage in CRS with eosinophilic infiltration. ECRS is linked to multiple biochemical signaling pathways that were wrongly thought to be completely separate from one another. In this paper, we developed a detailed and exhaustive characterization of all the main molecular signaling pathways involved in ECRS that explains the biochemical mechanisms behind clinical observations. Based on this characterization, this study developed (1) an electron delocalization chemical flowsheet of the mechanism of superoxide radical conversion into H_2_O_2_ by SOD and the subsequent breakdown of H_2_O_2_ into water and oxygen by catalase, (2) a biochemical flowsheet of the main molecular pathways involving CO and inflammation through CO’s modification of ERK, p38 and C/EBP δ protein production and function, and (3) a biochemical flowsheet of the IL-17A, HIF-1, NLRP3 inflammasome and IgE signaling pathways involved in the inflammatory processes described in this paper. Furthermore, based on this molecular characterization described in this study, all the main therapeutic targets for ECRS as a group and as a unified signaling pathway were identified for the first time as listed from (1) to (27): (1) HIF-1, (2) PTPN2, (3) Claudin-4, (4) Occludin, (5) ZO-1, (6) NLRP3, (7) IgE, (8) CO, (9) COX-2, (10) PGE-2, (11) p38, (12) C/EBP δ, (13) GSDMD, (14) IL-18, (15) IL-1β, (16) IL-2, (17) MEK, (18) ERK, (19) Catalase, (20) oxoferryl (IV) porphyrin radical cation, (21) H_2_O_2_, (22) Eotaxin-1, (23) NF-κB, (24) AP-1, (25) CREB, (26) IL-6 and (27) TNF-α. This group of therapeutic targets were identified for the first time based on the molecular characterization of the aforementioned signaling pathways developed in this study and the critical analysis of the scientific literature related to the following, listed from (I) to (VII): (I) Interleukin-17A (IL-17A), (II) superoxide dismutase (SOD), (III) heme-oxygenase-1 (HO-1), (IV) protein tyrosine phosphatase non-receptor type 2 (PTPN2), (V) NOD-like receptor protein 3 (NLRP3), (VI) the inflammasome and (VII) B cells. The overall conclusion is that antioxidants can play a critical role in elucidating the pathophysiological mechanisms of intractable diseases such as ECRS and could provide valuable therapeutic strategies.

## 11 Future direction

Current therapies for ECRS are associated with many unwanted side effects. Although various new biological therapies have been developed for ECRS and are sometimes covered by insurance, they remain subject to the inherent disadvantages associated with the disease. For example, recurrent nasal polyps are commonly observed in many cases following surgery for ECRS. That is why steroids remain the most commonly used conservative treatment. While steroids are effective, they come with a range of side effects, and long-term administration poses significant risks. However, discontinuing steroid treatment can lead to recurrence and enlargement of nasal polyps. Approximately 60% of patients with ECRS also have severe asthma, and it is not uncommon for these patients to already be on steroids for asthma management. In such cases, the steroid dosage may be further increased at the initiation of ECRS treatment. As per Nyugen et al. (140), although steroids are known to suppress eosinophil production, they can also promote the production of neutrophils, potentially exacerbating inflammation. Dupilumab, a human monoclonal antibody targeting the IL-4/13 receptor, has been available as a biological treatment for eosinophilic sinusitis and is covered by insurance. One of the reported side effects of dupilumab, according to Suzaki et al. (141), is eosinophilia and serious conditions like eosinophilic granulomatosis with polyangiitis have been linked to its use. This can lead to severe complications such as myocardial infarction, epicarditis, cerebral infarction, peripheral neuritis, gastrointestinal ulcers, and purpura. The high cost of dupilumab places a significant financial burden on patients and health systems, creating a strain on healthcare resources too.

In the biochemical aspect the present study contributed to a comprehensive characterization of the main therapeutic targets of ECRS as an extension of the study published by Joseph (142), which can help to better understand the pathogenesis of neurodegeneration such as in airway defense mechanisms reported in Sato et al. (143).

In the clinical aspect, the present study contributed to the development of safer and more cost-effective treatment options.

Based on the findings of both the biochemical and clinical facets in this study, we propose that targeting eosinophils, B cells and macrophages could be an effective treatment approach for ECRS.

Recent evidence further supports the importance of immune endotyping in chronic rhinosinusitis with nasal polyps (CRSwNP), particularly in the context of aspirin-exacerbated respiratory disease (AERD). Nazari et al. (144) demonstrated that patients with AERD and non-AERD CRSwNP exhibit distinct immune endotypes characterized by differential cytokine expression profiles, including variations in IL-17A levels and the degree of eosinophil infiltration. Their findings suggest that heterogeneity in cytokine milieu may directly influence disease severity and therapeutic responsiveness. The observation that IL-17A expression and eosinophil-driven inflammation differ between subgroups further reinforces the importance of molecular stratification in ECRS management. These results align with our integrative signaling model and highlight the potential value of endotype-based therapeutic selection in optimizing treatment outcomes for ECRS patients.

Further, similar integrative signaling models developed by our team regarding cancer apoptosis (cell death) are discussed in Joseph et al. (145).

## References

[1] Benninger MS FeDW Lanza DC Marple BF Osguthorpe JD Stankiewicz JA . Adult chronic rhinosinusitis: definitions, diagnosis, epidemiology, and pathophysiology. Otolaryngol Head Neck Surg. (2003) 129:S1–S32. doi: 10.1016/S0194-5998(03)01397-4 12958561

[2] Tsuda T Suzuki M Kato Y Kidoguchi M Kumai T Fjieda S . The current findings in eosinophilic chronic rhinosinusitis. Auris Nasus Larynx. (2024) 51:51–60. doi: 10.1016/j.anl.2023.08.002 37574421

[3] Oka A Kanai K Okano M . Pathogenesis and biomarkers of eosinophilic rhinosinusitis. Pract Otol(Kyoto). (2023) 116:405–11. doi: 10.5631/jibirin.116.289

[4] Fujieda S Imoto Y Kato Y Ninomiya T Tokunaga T Tsutumiuchi T . Eosinophilic chronic rhinosiusitis. Allergol Int. (2019) 68:403–12. doi: 10.2468/jbes.69.131 31402319

[5] Nonaka M Seo Y Sao E Mukai M Goto K . Characteristics of eosinophilic otitis media and eosinophilic chronic rhinosinusitis, and their relationships with comorbid asthma. Pract Otol(Kyoto). (2020) 113:405–11. doi: 10.5631/jibirin.113.405

[6] Chen Y Thai P Zhao YH Ho YS DeSouza MM Wu R . Stimulation of airway mucin gene expression by interleukin (IL)-17 through IL-6 paracrine/autocrine loop. J Biol Chem. (2003) 278:17036–43. doi: 10.1074/jbc.m210429200 12624114

[7] Poston RN Chanez P Lacoste JY Litchfield T Lee TH Bousquet J . Immunohistochemical characterization of the cellular infiltration in asthmatic bronchi. Am Rev Respir Dis. (1882) 145:918–21. doi: 10.1164/ajrccm/145.4_pt_1.918 1554221

[8] Seminario MC Gleich GJ . The role of eosinophils in the pathogenesis of asthma. Curr Opin Immunol. (1994) 6:860–4. doi: 10.1016/0952-7915(94)90005-1 7710710

[9] Vendrov AE Hakim ZS Madamanchi NR Rojas M Madamanchi C Runge MS . Atherosclerosis is attenuated by limiting superoxide generation in both macrophages and vessel wall cells. Arteriosclerosis Thrombosis Vasc Biol. (2007) 27:2714–21. doi: 10.1161/atvbaha.107.152629 17823367

[10] Iijima MK Kobayashi T Kamada H Shimojo N . Exposure to ozone aggravates nasal allergy-like symptoms in Guinea pigs. Toxicol Lett. (2001) 123:77–85. doi: 10.1016/s0378-4274(01)00392-7 11514108

[11] Makihara S Okano M Fujiwara T Kariya S Noda Y Higaki T . Regulation and characterization of IL-17A expression in patients with chronic rhinosinusitis and its relationship with eosinophilic inflammation. J Allergy Clin Immunol. (2010) 126:397–400. doi: 10.1016/j.jaci.2010.05.014 20621345

[12] Nishi T Maier CM Hayashi T Saito A Chan PH . Superoxide dismutase 1 overexpression reduces MCP-1 and MIP-1 alpha expression after transient focal cerebral ischemia. J Cereb Blood Flow Metab. (2005) 25:1312–24. doi: 10.1038/sj.jcbfm.9600124 15829914

[13] Saitoh T Kusunoki T Yao T Kawano K Kojima Y Miyahara K . Relationship between epithelial damage or basement membrane thickness and eosinophilic infiltration in nasal polyps with chronic rhinosinusitis. Rhinology. (2009) 47:275–9. doi: 10.4193/rhin08.190 19839250

[14] Saitoh T Kusunoki T Yao T Kawano K Kojima Y Miyahara K . Role of interleukin-17A in the eosinophil accumulation and mucosal remodeling in chronic rhinosinusitis with nasal polyps associated with asthma. Int Arch Allergy Immunol. (2010) 151:8–16. doi: 10.1159/000232566 19672092

[15] Ono N Kusunoki T Ikeda K . Relationships between IL-17A and macrophages or MUC5AC in pathological processes of eosinophilic chronic rhinosinusitis. Allergy Rhinol. (2012) 3:1–5. doi: 10.2500/ar.2012.3.0030 23342289 PMC3548608

[16] Ono N Kusunoki T Miwa M Hitotsu M Shiozawa A Ikeda K . Reduction in superoxide disumutase expression in the mucosa of eosinophilic chronic rhinosinusitis with nasal polyps. In Arch Allergy Immunol. (2013) 162:75–82. doi: 10.1159/000353122 23921602

[17] Kusunoki T Ono N Ikeda K . Cu,Zn-superoxide dismutase and macrophage or MUC5AC in human eosinophilic chronic rhinosinusitis. J Otol Rhinol. (2015) S1:1.

[18] Tagaya E Tamaoki J . Mechanisms of airway remodeling in asthma. Allergol Int. (2007) 56:311–40. doi: 10.2332/allergolint.r-07-152 17965576

[19] Cundall M Sun Y Miranda C Trueau JB Bames S . Neutrophil-derived matrix metalloproteinase-9 is increased in severe asthma and poorly inhibited by glucocoricoids. J Allergy Clin Immunol. (2003) 112:1064–71. doi: 10.1016/j.jaci.2003.08.013 14657859

[20] Wenzel SE Schwartz LB Langmack EL Halliday JL Trudeau JB . Evidence that severe asthma can be divided pathologically into two inflammatory subtypes with distinct physiologic and clinical characteristics. Am J Respir Crit Care Med. (1999) 160:1001–8. doi: 10.1164/ajrccm.160.3.9812110 10471631

[21] Martney JM Gustason CE Bowler RP Pelanda R Torres RM . Thromboxane receptor signaling is required for fibronectin-induced matrix metalloproteinase-9 production by human and murine macrophages and is attenuated by Arhgel1 molecule. J Biol Chme. (2011) 286:44521–31. doi: 10.1074/jbc.M111.282772 22086927 PMC3247948

[22] Sawatsubashi M . Understanding the key issue in the diagnosis and treatment for type 2 chronic rhinosinusitis. Otol Fukuoka. (2024) 70:32–43.

[23] Fokkens WJ Lund VJ Hopkins C Hellings PW Kern R Reitsma S . European position paper on rhinosiustis and nasal polyps 2020. Rhinology. (2020) 58:1–464. doi: 10.4193/rhin20.600 32077450

[24] Haruna S . Eosinophilic sinusitis. ORL Tokyo. (2001) 44:195–201.

[25] Tokunaga T Sakashita M Haruna T Asaka D Takeno S Ikeda H . Novel scoring system and algorithm for classifying chronic rhinosinusitis-The JESREC Study-. Allergy. (2015) 70:995–1003. 25945591 10.1111/all.12644 PMC5032997

[26] Giunta G Pirola F Giombi F Muci G Pace GM Heffler E . Care for patients with type-e chronic rhinosinusitis. J Pwrs Med. (2023) 13:618. doi: 10.3390/jpm13040618 37109003 PMC10146372

[27] Cho SH Hamilos DL Han DH Laidaw TM . Phenotypes of chronic rhinosinusitis. J Allergy Clin Immunol Pract. (2020) 8:1505–11. doi: 10.1016/j.jaip.2019.12.021 32389275 PMC7696652

[28] Akdis CA Bachert C Cingi C Dykewicz MS Hellings PW Naclerio RM . Entotypes and phenotypes of the European academy of allergy and clinical immunology and the American academy of allergy. Athma Immunol. (2013) 131:1479–90. doi: 10.1016/j.jaci.2013.02.036 23587334 PMC4161279

[29] Bachert C Zhang N . Medical algorithm-diagnosis and treatment of chronic rhinosinusitis. Allergy. (2020) 75:240–2. doi: 10.1111/all.13823 30993703

[30] Bachert C Zhang N Cavaliere C Weiping W Gevaert E Krysko O . Biologics for chronic rhinosinusitis with nasal polyps. J Allergy Clin Immunol. (2020) 145:725–39. doi: 10.1016/j.jaci.2020.01.020 32145872

[31] Jovanovic DV Di Battista JA Martel-Pelletier J Jolicoeur FC He Y Zhang M . IL-17 stimulates the production and expression of proinflammatory cytokines, IL-β and TNF-α, by human macrophages. J Immunol. (1998) 160:3513–21. doi: 10.4049/jimmunol.160.7.3513 9531313

[32] Rahman MS Yamasaki A Yang J Shan L Halayko AJ Gounni AS . IL-17A induces eotaxin-1/CC chemokine ligand 11 expression in human airway smooth muscle cells: role of MAPK (Erk1/2, JNK, and p38) pathways. J Immunol. (2006) 177:4064–71. doi: 10.4049/jimmunol.177.6.4064 16951370

[33] Ivanovska M Abdi Z Murdjeva M Macedo D Maes A Maes M . CCL-11 or eotaxin-1: an immune marker for ageing and accelerated ageing in neuro-psychiatric disorders. Pharmaceuticals. (2020) 13:230. doi: 10.3390/ph13090230 32887304 PMC7558796

[34] Huang G Wang Y Vogel P Chi H . Control of IL-17 receptor signaling and tissue inflammation by the p38α–MKP-1 signaling axis in a mouse model of multiple sclerosis. Sci Signaling. (2015) 8:ra24–4. doi: 10.1126/scisignal.aaa2147 25737586 PMC4640462

[35] Sawant KV Poluri KM Dutta AK Sepuru KM Troshkina A Garofalo RP . Chemokine CXCL1 mediated neutrophil recruitment: role of glycosaminoglycan interactions. Sci Rep. (2016) 6:33123. doi: 10.1038/srep33123 27625115 PMC5021969

[36] Tierney JW Evans BC Cheung-Flynn J Wang B Colazo JM Polcz ME . Therapeutic MK2 inhibition blocks pathological vascular smooth muscle cell phenotype switch. JCI Insight. (2021) 6:e142339. doi: 10.1172/jci.insight.142339 34622803 PMC8525639

[37] Tai J Shin JM Park J Han M Kim TH . Oxidative stress and antioxidants in chronic rhinosinusitis with nasal polyps. Antioxidants. (2023) 12:195. doi: 10.3390/antiox12010195 36671057 PMC9854928

[38] Zheng M Liu Y Zhang G Yang Z Xu W Chen Q . The application and mechanisms of superoxide dismutase in medicine, food, and cosmetics. Antioxidants. (2023) 12:1675. doi: 10.3390/antiox12091675 37759978 PMC10525108

[39] Pae HO Lee YC Chung HT . Heme oxygenase-1 and carbon monoxide: emerging therapeutic targets in inflammation and allergy. Recent Patents Inflammation Allergy Drug Discov. (2008) 2:159–65. doi: 10.2174/187221308786241929 19076005

[40] Hauber HP Goldmann T Vollmer E Wollenberg B Zabel P . Effect of dexamethasone and ACC on bacteria-induced mucin expression in human airway mucosa. Am J Respir Cell Mol Biol. (2007) 37:606–16. doi: 10.1165/rcmb.2006-0404oc 17600317

[41] Tainer JA Getzoff ED Richardson JS Richardson DC . Structure and mechanism of copper, zinc superoxide dismutase. Nature. (1983) 306:284–7. doi: 10.1038/306284a0 6316150

[42] Fita I Rossmann MG . The active center of catalase. J Mol Biol. (1985) 185:21–37. doi: 10.1016/0022-2836(85)90180-9 4046038

[43] Kato S Ueno T Fukuzumi S Watanabe Y . Catalase reaction by myoglobin mutants and native catalase: mechanistic investigation by kinetic isotope effect. J Biol Chem. (2004) 279:52376–81. doi: 10.1074/jbc.m403532200 15347658

[44] Goyal MM Basak A . Human catalase: looking for complete identity. Protein Cell. (2010) 1:888–97. doi: 10.1007/s13238-010-0113-z 21204015 PMC4875117

[45] Pan Z Zhang R Newcomb M . Kinetic studies of reactions of iron (IV)-oxo porphyrin radical cations with organic reductants. J Inorg Biochem. (2006) 100:524–32. doi: 10.1016/j.jinorgbio.2005.12.022 16500709

[46] Putnam CD Arvai AS Bourne Y Tainer JA . Active and inhibited human catalase structures: ligand and NADPH binding and catalytic mechanism. J Mol Biol. (2000) 296:295–309. doi: 10.1006/jmbi.1999.3458 10656833

[47] Joseph D . The fundamental neurobiological mechanism of oxidative stress-related 4E-BP2 protein deamidation. Int J Mol Sci. (2024) 25:12268. doi: 10.3390/ijms252212268 39596333 PMC11594350

[48] Ponaka P . Cell billogy of heme. Am J Med Sci. (199) 318:241–56. 10.1097/00000441-199910000-00004 10522552

[49] Choi AMK Alam L . Heme oxygenase-1: function, regulation, and implication of a novel stress-inducible protein in oxidant-induced lung injury. Am J Respir Cell Mol Biol. (1996) 15:9–19. doi: 10.1165/ajrcmb.15.1.8679227 8679227

[50] Hovath I Loukides S Wodelhouse T Khartonov SA Cole PJ Baranes PJ . Increased levels of exhaled carbon monoxide in bronchiectasis: a new marker of oxidative stress. Thorax. (1998) 53:867–70. doi: 10.1136/thx.53.10.867 10193374 PMC1745084

[51] Elhini A Abdelwanhab S Ikeda K . Heme oxygenase(HO)-1 is upregulated in the nasal mucosa with allergic rhinitis. Laryngoscope. (2006) 119:446–50. doi: 10.1097/01.mlg.0000194692.51979.d1 16540907

[52] Kawano K Kusunoki T Ono N Yao T Saito T Yokoi H . Heme oxygenase-1 expression in chronic rhinosinusitis with eosinophilic infiltration. Anl. (2012) 39:387–92. doi: 10.1016/j.anl.2011.10.001 22078849

[53] Otterbein LE Bach FH Alam J Soares M Tao Lu H Wysk M . Carbon monoxide has anti-inflammatory effects involving the mitogen-activated protein kinase pathway. Nat Med. (2000) 6:422–8. doi: 10.1038/74680 10742149

[54] Ryter SW Alam J Choi AM . Heme oxygenase-1/carbon monoxide: from basic science to therapeutic applications. Physiol Rev. (2006) 86:583–650. doi: 10.1152/physrev.00011.2005 16601269

[55] Hambleton J Weinstein SL Lem L DeFranco AL . Activation of c-Jun N-terminal kinase in bacterial lipopolysaccharide-stimulated macrophages. Proc Natl Acad Sci. (1996) 93:2774–8. doi: 10.1073/pnas.93.7.2774 8610116 PMC39708

[56] Raingeaud J Gupta S Rogers JS Dickens M Han J Ulevitch RJ . Pro-inflammatory cytokines and environmental stress cause p38 mitogen-activated protein kinase activation by dual phosphorylation on tyrosine and threonine (∗). J Biol Chem. (1995) 270:7420–6. doi: 10.1074/jbc.270.13.7420 7535770

[57] Otterbein LE Otterbein SL Ifedigbo E Liu F Morse DE Fearns C . MKK3 mitogen-activated protein kinase pathway mediates carbon monoxide-induced protection against oxidant-induced lung injury. Am J Pathol. (2003) 163:2555–63. doi: 10.1016/s0002-9440(10)63610-3 14633627 PMC1892354

[58] Toribio ML Gutiérrez-Ramos JC Pezzi L Marcos MA Martínez-A C . lnterleukin-2-dependent autocrine proliferation in T-cell development. Nature. (1989) 342:82–5. doi: 10.1038/342082a0 2812004

[59] DeSilva DR Jones EA Favata MF Jaffee BD Magolda RL Trzaskos JM . Inhibition of mitogen-activated protein kinase kinase blocks T cell proliferation but does not induce or prevent anergy. J Immunol. (1998) 160:4175–81. doi: 10.4049/jimmunol.160.9.4175 9574517

[60] Pae HO Oh GS Choi BM Chae SC Kim YM Chung KR . Carbon monoxide produced by heme oxygenase-1 suppresses T cell proliferation via inhibition of IL-2 production. J Immunol. (2004) 172:4744–51. doi: 10.4049/jimmunol.172.8.4744 15067050

[61] Chandrasekharan NV Simmons DL . The cyclooxygenases. Genome Biol. (2004) 5:1–7. doi: 10.1186/gb-2004-5-9-241 15345041 PMC522864

[62] Yin Y Wang J Li J . A concise and scalable chemoenzymatic synthesis of prostaglandins. Nat Commun. (2024) 15:2523. doi: 10.1038/s41467-024-46960-y 38514642 PMC10957970

[63] Williams TJ Peck MJ . Role of prostaglandin-mediated vasodilatation in inflammation. Nature. (1977) 270:530–2. doi: 10.1038/270530a0 593374

[64] Soares CLR Wilairatana P Silva LR Moreira PS Barbosa NMMV da Silva PR . Biochemical aspects of the inflammatory process: A narrative review. Biomed Pharmacother. (2023) 168:115764. doi: 10.1016/j.biopha.2023.115764 37897973

[65] Wang D Cabalag CS Clemons NJ DuBois RN . Cyclooxygenases and prostaglandins in tumor immunology and microenvironment of gastrointestinal cancer. Gastroenterology. (2021) 161:1813–29. doi: 10.1053/j.gastro.2021.09.059 34606846

[66] Suh GY Jin Y Yi AK Wang XM Choi AM . CCAAT/enhancer-binding protein mediates carbon monoxide–induced suppression of cyclooxygenase-2. Am J Respir Cell Mol Biol. (2006) 35:220–6. doi: 10.1165/rcmb.2005-0154oc 16543610 PMC2643257

[67] Chen JJ Huang WC Chen CC . Transcriptional regulation of cyclooxygenase-2 in response to proteasome inhibitors involves reactive oxygen species-mediated signaling pathway and recruitment of CCAAT/enhancer-binding protein δ and CREB-binding protein. Mol Biol Cell. (2005) 16:5579–91. doi: 10.1091/mbc.e05-08-0778 16195339 PMC1289404

[68] Ejima K Layne MD Carvajal IM Kritek PA Baron RM Chen YH . Cyclooxygenase‐2 deficient mice are resistant to endotoxin‐induced inflammation and death. FASEB J. (2003) 17:1325–7. doi: 10.1096/fj.02-1078fje 12738799

[69] Wang GL Semenza GL . Purification and characterization of hypoxia-inducible factor 1 (∗). J Biol Chem. (1995) 270:1230–7. doi: 10.1074/jbc.270.3.1230 7836384

[70] O'Rourke JF Dachs GU Gleadle JM Maxwell PH Pugh CW Stratford IJ . Hypoxia response elements. Oncol Res. (1997) 9:327–32. 9406238

[71] Chien CY Tai CF Ho KY Kuo WR Chai CY Hsu YC . Expression of hypoxia-inducible factor 1a in the nasal polyps by real-time RT-PCR and immunohistochemistry. Otolaryngol—Head Neck Surg. (2008) 139:206–10. doi: 10.1016/j.otohns.2008.04.022 18656716

[72] Otani T Furuse M . Tight junction structure and function revisited. Trends Cell Biol. (2020) 30:805–17. doi: 10.1016/j.tcb.2020.08.004 32891490

[73] Sugita K Kabashima K . Tight junctions in the development of asthma, chronic rhinosinusitis, atopic dermatitis, eosinophilic esophagitis, and inflammatory bowel diseases. J Leukocyte Biol. (2020) 107:749–62. doi: 10.1002/jlb.5mr0120-230r 32108379

[74] Zhang M Xiong Y Tu J Tang B Zhang Z Yu J . Hypoxia disrupts the nasal epithelial barrier by inhibiting PTPN2 in chronic rhinosinusitis with nasal polyps. Int Immunopharmacol. (2023) 118:110054. doi: 10.1016/j.intimp.2023.110054 36963262

[75] Laidlaw TM Prussin C Panettieri RA Lee S Ferguson BJ Adappa ND . Dexpramipexole depletes blood and tissue eosinophils in nasal polyps with no change in polyp size. Laryngoscope. (2019) 129:E61–6. doi: 10.1002/lary.27564 30284267

[76] Buchheit KM Dwyer DF Ordovas-Montanes J Katz HR Lewis E Vukovic M . IL-5Rα marks nasal polyp IgG4-and IgE-expressing cells in aspirin-exacerbated respiratory disease. J Allergy Clin Immunol. (2020) 145:1574–84. doi: 10.1016/j.anai.2023.05.016 32199912 PMC7282948

[77] Schwitzguébel AJP Jandus P Lacroix JS Seebach JD Harr T . Immunoglobulin deficiency in patients with chronic rhinosinusitis: systematic review of the literature and meta-analysis. J Allergy Clin Immunol. (2015) 136:1523–31. doi: 10.1016/j.jaci.2015.07.016 26329513

[78] Tan BK Li QZ Suh L Kato A Conley DB Chandra RK . Evidence for intranasal antinuclear autoantibodies in patients with chronic rhinosinusitis with nasal polyps. J Allergy Clin Immunol. (2011) 128:1198–206. doi: 10.1016/j.jaci.2011.08.037 21996343 PMC3384688

[79] Gould HJ Sutton BJ . IgE in allergy and asthma today. Nat Rev Immunol. (2008) 8:205–17. doi: 10.1038/nri2273 18301424

[80] Zhang N Holtappels G Gevaert P Patou J Dhaliwal B Gould H . Mucosal tissue polyclonal IgE is functional in response to allergen and SEB. Allergy. (2011) 66:141–8. doi: 10.1111/j.1398-9995.2010.02448.x 20659077

[81] Moon TC Befus AD Kulka M . Mast cell mediators: their differential release and the secretory pathways involved. Front Immunol. (2014) 5:569. doi: 10.3389/fimmu.2014.00569 25452755 PMC4231949

[82] López JC . Histamine's comeback? Nat Rev Neurosci. (2002) 3:84. doi: 10.1038/nrn737 37880705

[83] Gevaert P Omachi TA Corren J Mullol J Han J Lee SE . Efficacy and safety of omalizumab in nasal polyposis: 2 randomized phase 3 trials. J Allergy Clin Immunol. (2020) 146:595–605. doi: 10.1016/j.jaci.2020.05.032 32524991

[84] Kaplan AP Gimenez‐Arnau AM Saini SS . Mechanisms of action that contribute to efficacy of omalizumab in chronic spontaneous urticaria. Allergy. (2017) 72:519–33. doi: 10.1111/all.13083 27861988 PMC5915348

[85] Li Y Chang LH Huang WQ Bao HW Li X Chen XH . IL-17A mediates pyroptosis via the ERK pathway and contributes to steroid resistance in CRSwNP. J Allergy Clin Immunol. (2022) 150:337–51. doi: 10.1016/j.jaci.2022.02.031 35346673

[86] Ghonime MG Shamaa OR Das S Eldomany RA Fernandes-Alnemri T Alnemri ES . Inflammasome priming by lipopolysaccharide is dependent upon ERK signaling and proteasome function. J Immunol. (2014) 192:3881–8. doi: 10.4049/jimmunol.1301974 24623131 PMC3980013

[87] Compan V Martín-Sánchez F Baroja-Mazo A López-Castejón G Gomez AI Verkhratsky A . Apoptosis-associated speck-like protein containing a CARD forms specks but does not activate caspase-1 in the absence of NLRP3 during macrophage swelling. J Immunol. (2015) 194:1261–73. doi: 10.4049/jimmunol.1301676 25552542 PMC4883655

[88] Bai Y Pan Y Liu X . Mechanistic insights into gasdermin-mediated pyroptosis. Nat Rev Mol Cell Biol. (2025) 26:501–21. doi: 10.1038/s41580-025-00837-0 40128620

[89] Head K Chong LY Hopkins C Philpott C Burton MJ Schilder AG . Short‐course oral steroids alone for chronic rhinosinusitis. Cochrane Database Systematic Rev. (2016) 2016:CD011991. doi: 10.1002/14651858.cd011991 27113367 PMC8504433

[90] Lin H Li Z Lin D Zheng C Zhang W . Role of NLRP3 inflammasome in eosinophilic and non-eosinophilic chronic rhinosinusitis with nasal polyps. Inflammation. (2016) 39:2045–52. doi: 10.1007/s10753-016-0442-z 27614764

[91] Creangă EC Ott C Nicolae AC Drăgoi CM Stan R . Roxadustat as a hypoxia-mimetic agent: Erythropoietic mechanisms, bioanalytical detection, and regulatory considerations in sports medicine. Curr Issues Mol Biol. (2025) 47:734. doi: 10.3390/cimb47090734 41020856 PMC12468147

[92] Mullard A . FDA approves first hypoxia-inducible factor prolyl hydroxylase inhibitor. Nat Rev Drug Discov. (2023) 22:173. doi: 10.1038/d41573-023-00028-6 36759582

[93] Chertow GM Eckardt KU Sarnak MJ Winkelmayer WC Agarwal R Minga T . Safety and efficacy of vadadustat for the treatment of CKD-related anemia within and outside the United States. J Am Soc Nephrol. (2025) 36:1984–97. doi: 10.1681/ASN.0000000708 40359056 PMC12499611

[94] Joharapurkar A Pandya V Patel H Jain M Desai R . Desidustat: a novel PHD inhibitor for the treatment of CKD-induced anemia. Front Nephrol. (2024) 4:1459425. doi: 10.3389/fneph.2024.1459425 39502472 PMC11534831

[95] Markham A . Enarodustat: first approval. Drugs. (2021) 81:169–74. doi: 10.1007/s40265-020-01444-3 33320297

[96] Lawson H Holt-Martyn JP Dembitz V Kabayama Y Wang LM Bellani A . The selective prolyl hydroxylase inhibitor IOX5 stabilizes HIF-1α and compromises development and progression of acute myeloid leukemia. Nat Cancer. (2024) 5:916–37. doi: 10.1038/s43018-024-00761-w 38637657 PMC11208159

[97] Arenillas C Toledo RA . FDA fast-track approval of belzutifan is a milestone in rare cancer therapy. Nat Rev Endocrinol. (2025) 21:739–40. doi: 10.1038/s41574-025-01183-z 40935878

[98] Baumgartner CK Ebrahimi-Nik H Iracheta-Vellve A Hamel KM Olander KE Davis TG . The PTPN2/PTPN1 inhibitor ABBV-CLS-484 unleashes potent anti-tumour immunity. Nature. (2023) 622:850–62. doi: 10.1038/s41586-023-06575-7 37794185 PMC10599993

[99] Morón B Spalinger M Kasper S Atrott K Frey-Wagner I Fried M . Activation of protein tyrosine phosphatase non-receptor type 2 by spermidine exerts anti-inflammatory effects in human THP-1 monocytes and in a mouse model of acute colitis. PloS One. (2013) 8:e73703. doi: 10.1371/journal.pone.0073703 24040033 PMC3767590

[100] Li E Li C Horn N Ajuwon KM . Quercetin attenuates deoxynivalenol-induced intestinal barrier dysfunction by activation of Nrf2 signaling pathway in IPEC-J2 cells and weaned piglets. Curr Res Toxicol. (2023) 5:100122. doi: 10.1016/j.crtox.2023.100122 37720305 PMC10500468

[101] Xu Y Ou J Zhang C Chen J Chen J Li A . Rapamycin promotes the intestinal barrier repair in ulcerative colitis via the mTOR/PBLD/AMOT signaling pathway. Biochim Biophys Acta (BBA)-Molecular Basis Dis. (2024) 1870:167287. doi: 10.1016/j.bbadis.2024.167287 38862095

[102] Coll RC Hill JR Day CJ Zamoshnikova A Boucher D Massey NL . MCC950 directly targets the NLRP3 ATP-hydrolysis motif for inflammasome inhibition. Nat Chem Biol. (2019) 15:556–9. doi: 10.1038/s41589-019-0277-7 31086327

[103] Choi H-I Alam Zeb Kim M-S Rana I Khan N Qureshi OS . Controlled therapeutic delivery of CO from carbon monoxide-releasing molecules (CORMs). J Controlled Release. (2022) 350:652–67. doi: 10.1016/j.jconrel.2022.08.055 36063960

[104] Hayashi K Miki K Ueno H Sekiguchi M Arai YC Nikaido T . Randomized open-label non-inferiority trial of paracetamol or celecoxib for patients with chronic low back pain. Sci Rep. (2026) 16:1577. doi: 10.1038/s41598-025-31094-y 41366055 PMC12800242

[105] Flower RJ Vane JR . Inhibition of prostaglandin synthetase in brain explains the anti-pyretic activity of paracetamol (4-acetamidophenol). Nature. (1972) 240:410–1. doi: 10.1038/240410a0 4564318

[106] Miller C Humphreys IM Davis GE . Effect of over the counter ibuprofen dosing after sinus surgery for chronic rhinosinusitis: a prospective cohort pilot study. Ann Otol Rhinol Laryngol. (2020) 129:677–83. doi: 10.1177/0003489420906179 32028782 PMC10163897

[107] Murphy R . Extra-virgin olive oil has similar activity to ibuprofen. Nat Clin Pract Rheumatol. (2005) 1:66. doi: 10.1038/ncprheum0023 37880705

[108] Rouzer CA Marnett LJ . Cyclooxygenases: structural and functional insights. J Lipid Res. (2009) 50:S29–34. doi: 10.1194/jlr.r800042-jlr200 18952571 PMC2674713

[109] Schwenger P Bellosta P Vietor I Basilico C Skolnik EY Vilček J . Sodium salicylate induces apoptosis via p38 mitogen-activated protein kinase but inhibits tumor necrosis factor-induced c-Jun N-terminal kinase/stress-activated protein kinase activation. Proc Natl Acad Sci. (1997) 94:2869–73. doi: 10.1073/pnas.94.7.2869 9096313 PMC20289

[110] Chang L-H Huang H-S Wu P-T Jou I-M Pan M-H Chang W-C . Role of macrophage CCAAT/Enhancer binding protein delta in the pathogenesis of rheumatoid arthritis in collagen-induced arthritic mice. PloS One. (2012) 7:e45378. doi: 10.1371/journal.pone.0045378 23028973 PMC3454428

[111] Boxer MB Shen M Auld DS Wells JA Thomas CJ . A small molecule inhibitor of Caspase 1. In: . National Center for Biotechnology Information (US, Bethesda, MD (2011). Available online at: https://www.ncbi.nlm.nih.gov/books/NBK56247/. 21735610

[112] Sageshima J Ciancio G Chen L Burke GW . Anti-interleukin-2 receptor antibodies—basiliximab and daclizumab—for the prevention of acute rejection in renal transplantation. Biol: Targets Ther. (2009) 3:319–36. doi: 10.2147/btt.2009.3257 19707418 PMC2726067

[113] Grimaldi AM Simeone E Festino L Vanella V Strudel M Ascierto PA . MEK inhibitors in the treatment of metastatic melanoma and solid tumors. Am J Clin Dermatol. (2017) 18:745–54. doi: 10.1007/s40257-017-0292-y 28537004

[114] Xiao H Wang A Shuai W Qian Y Wu C Wang X . A first-in-class selective inhibitor of ERK1/2 and ERK5 overcomes drug resistance with a single-molecule strategy. Signal Transduction Targeted Ther. (2025) 10:70. doi: 10.1038/s41392-025-02169-z 39979271 PMC11842588

[115] Lin X Bai D Wei Z Zhang Y Huang Y Deng H . Curcumin attenuates oxidative stress in RAW264. 7 cells by increasing the activity of antioxidant enzymes and activating the Nrf2-Keap1 pathway. PloS One. (2019) 14:e0216711. doi: 10.1371/journal.pone.0216711 31112588 PMC6528975

[116] Suzuki Y Hada M Fujii H . Synthesis, characterization, and reactivity of oxoiron (IV) porphyrin π-cation radical complexes bearing cationic N-methyl-2-pyridinium group. J Inorg Biochem. (2021) 223:111542. doi: 10.1016/j.jinorgbio.2021.111542 34293682

[117] Karakioulaki M Eyerich K Patsatsi A . Advancements in bullous pemphigoid treatment: a comprehensive pipeline update. Am J Clin Dermatol. (2024) 25:195–212. doi: 10.1007/s40257-023-00832-1 38157140 PMC10866767

[118] Kusaki M Ohta Y Inufusa H Yamashita T Morihara R Nakano Y . Neuroprotective effects of a novel antioxidant mixture Twendee X in mouse stroke model. J Stroke Cerebrovascular Dis. (2017) 26:1191–6. doi: 10.1016/j.jstrokecerebrovasdis.2017.01.003 28190603

[119] Liu X Yamashita T Shang J Shi X Morihara R Huang Y . Clinical and pathological benefit of Twendee X in Alzheimer's disease transgenic mice with chronic cerebral hypoperfusion. J Stroke Cerebrovascular Dis. (2019) 28:1993–2002. doi: 10.1016/j.jstrokecerebrovasdis.2019.03.029 31029568

[120] Tadokoro K Morihara R Ohta Y Hishikawa N Kawano S Sasaki R . Clinical benefits of antioxidative supplement Twendee X for mild cognitive impairment: A multicenter, randomized, double-blind, and placebo-controlled prospective interventional study. J Alzheimer’s Dis. (2019) 71:1063–9. doi: 10.3233/jad-190644 31476161

[121] Liu X Yamashita T Shang J Shi X Morihara R Huang Y . Twendee X ameliorates phosphorylated tau, α-synuclein and neurovascular dysfunction in Alzheimer's disease transgenic mice with chronic cerebral hypoperfusion. J Stroke Cerebrovascular Dis. (2019) 28:104310. doi: 10.1016/j.jstrokecerebrovasdis.2019.104310 31383622

[122] Tadokoro K Ohta Y Inufusa H Loon AFN Abe K . Prevention of cognitive decline in Alzheimer's disease by novel antioxidative supplements. Int J Mol Sci. (2020) 21:1974. doi: 10.3390/ijms21061974 32183152 PMC7139972

[123] Feng T Yamashita T Tsunoda K Matsumoto N Tadokoro K Sasaki R . *In vitro* free radical scavenging activities of dietary supplements by electron spin resonance. Brain Supplement. (2020) 2:1–12. doi: 10.57361/brainsupplement.2.0_1 42241809

[124] Hu X Yamashita T Yu H Bian Z Hu X Feng T . Neuroprotective and therapeutic effects of Tocovid and Twendee-X on Aβ oligomer-induced damage in the SH-SY5Y cell line. Neurodegenerative Dis. (2021) 21:117–25. doi: 10.1159/000523983 35272285

[125] You F Tanaka S Yoshikawa T Matuschka von Greiffenclau M Inufusa H . Effects of antioxidant composition Twendee X on side effects of SARS-CoV-2 mRNA vaccine. Brain Supplement. (2022) 4:1–6. doi: 10.57361/brainsupplement.4.0_1 42241809

[126] You F Tanaka S Yoshikawa T Matuschka von Greiffenclau M Inufusa H . Antioxidant composition Twendee X may improve long COVID symptoms. Brain Supplement. (2022) 4:7–12. doi: 10.57361/brainsupplement.4.0_7 42241809

[127] Fukui K You F Kato Y Kimura M Harakawa Y Yoshikawa T . Twendee X, a mixed antioxidant supplement, improves cognitive function, coordination, and neurotrophic factor expression in long-term vitamin E-deficient mice. J Clin Biochem Nutr. (2023) 72:93–100. doi: 10.3164/jcbn.22-55 36936879 PMC10017315

[128] You F Harakawa Y Inufusa H . Considering antioxidant supplements as a means to prevent diseases. J Neurosci Neurol Disord. (2023) 7:14–6. doi: 10.29328/journal.jnnd.1001075

[129] Hirano S Inufusa H You F Sugiyama Y Kaneko M Yoshikawa T . Anti-oxidant Twendee X for maintenance of singing voice. Brain Supplement. (2023) 5:1–7. doi: 10.57361/brainsupplement.5.0_1 42241809

[130] You F Harakawa Y Yoshikawa T Inufusa H . Why does the antioxidant complex Twendee X® prevent dementia? Int J Mol Sci. (2023) 24:13018. doi: 10.20944/preprints202307.2112.v1 37629197 PMC10455760

[131] You F Harakawa Y Yoshikawa T Inufusa H . Potential of an antioxidant combination Twendee X® to treat depressive disorders. Arch Depression Anxiety. (2023) 9:68–71. doi: 10.17352/2455-5460.000083

[132] You F Harakawa Y Yoshikawa T Inufusa H . Controlling gut microbiota by Twendee X® may contribute to dementia prevention. Int J Mol Sci. (2023) 24:16642. doi: 10.3390/ijms242316642 38068966 PMC10706060

[133] Hirano S Inufusa H You F . The effect of oxidative stress on the human voice. Int J Mol Sci. (2024) 25:2604. doi: 10.3390/ijms25052604 38473848 PMC10932051

[134] Fukui K You F Kato Y Yuzawa S Kishimoto A Hara T . A blended vitamin supplement improves spatial cognitive and short-term memory in aged mice. Int J Mol Sci. (2024) 25:2804. doi: 10.3390/ijms25052804 38474050 PMC10932377

[135] Fukui K You F Kato Y Yuzawa S Kishimoto A Hara T . A mixed antioxidant supplement improves cognitive function and coordination in aged mice. J Clin Biochem Nutr. (2024) 74:119–26. doi: 10.3164/jcbn.23-71 38510681 PMC10948352

[136] You F Nicco C Harakawa Y Yoshikawa T Inufusa H . The potential of Twendee X® as a safe antioxidant treatment for systemic sclerosis. Int J Mol Sci. (2024) 25:3064. doi: 10.3390/ijms25053064 38474309 PMC10932212

[137] Miller SC Huang R Sakamuru S Shukla SJ Attene-Ramos MS Shinn P . Identification of known drugs that act as inhibitors of NF-κB signaling and their mechanism of action. Biochem Pharmacol. (2010) 79:1272–80. doi: 10.1016/j.bcp.2009.12.021 20067776 PMC2834878

[138] Ye N Ding Y Wild C Shen Q Zhou J . Small molecule inhibitors targeting activator protein 1 (AP-1) miniperspective. J Med Chem. (2014) 57:6930–48. doi: 10.1021/jm5004733 24831826 PMC4148154

[139] Steven A Friedrich M Jank P Heimer N Budczies J Denkert C . What turns CREB on? And off? And why does it matter? Cell Mol Life Sci. (2020) 77:4049–67. doi: 10.1007/s00018-020-03525-8 32347317 PMC7532970

[140] Nyugen LT Lim S Chung KF . Increase in airway neutrophils after oral but not inhaled corticosteroid therapy in mild asthama. Respirory Med. (2005) 99:200–7. doi: 10.1016/j.rmed.2004.06.007 15715187

[141] Suzaki K Tanaka A Yanai R Maruyama Y Kamimura S Hirano K . Eosinophilic granulomatosis with polyangiitis developed after dupolumab administration in patients with eosinophilic chronic rhinnosinusitis and asthma: a case report. BMC Pulmonary Med. (2023) 23:130. doi: 10.1186/s12890-023-02415-6 37076824 PMC10114392

[142] Joseph D . The unified theory of neurodegeneration pathogenesis based on axon deamidation. Int J Mol Sci. (2025) 26:4143. doi: 10.3390/ijms26094143 40362380 PMC12071446

[143] Sato Y Sugiyama Y Ishida T Inufusa H You F Joseph D . The potential role of oxidative stress in modulating airway defensive reflexes. Antioxidants. (2025) 14:568. doi: 10.3390/antiox14050568 40427451 PMC12108395

[144] Nazari J Shahba F Jafariaghdam N Mohebbi S Arshi S Bemanian MH . Immune endotyping and gene expression profile of patients with chronic rhinosinusitis with nasal polyps in the aspirin-exacerbated respiratory disease (AERD) and the non-AERD subgroups. Allergy Asthma Clin Immunol. (2024) 20:14. doi: 10.1186/s13223-024-00876-w 38360807 PMC10870654

[145] Joseph D Kongoli F You F . A unified therapeutic theory for treating cancer via master regulators of the universal apoptosis network. Cell Death Discov. (2026) 12:213. doi: 10.1038/s41420-026-03066-2 41922312 PMC13168256

